# Electrochemically Prepared Polyaniline as an Alternative to Poly(3,4-ethylenedioxythiophene)-poly(styrenesulfonate) for Inverted Perovskite Solar Cells

**DOI:** 10.1021/acsaem.2c00621

**Published:** 2022-07-20

**Authors:** Sally Mabrouk, Ashim Gurung, Behzad Bahrami, Abiral Baniya, Raja Sekhar Bobba, Fan Wu, Rajesh Pathak, Quinn Qiao

**Affiliations:** † Mechanical and Aerospace Engineering, Syracuse University, Syracuse, New York 13244, United States; ‡ Center for Advanced Photovoltaics, Department of Electrical Engineering and Computer Science, South Dakota State University, Brookings, South Dakota 57007, United States; § Key Lab of Optoelectronic Materials and Devices, School of Science, Huzhou University, Huzhou 313000, China

**Keywords:** inverted perovskite solar cells, hole transport layer, conducting polymers, PEDOT:PSS, PANI, electrochemical synthesis, doping, conductivity and work function

## Abstract

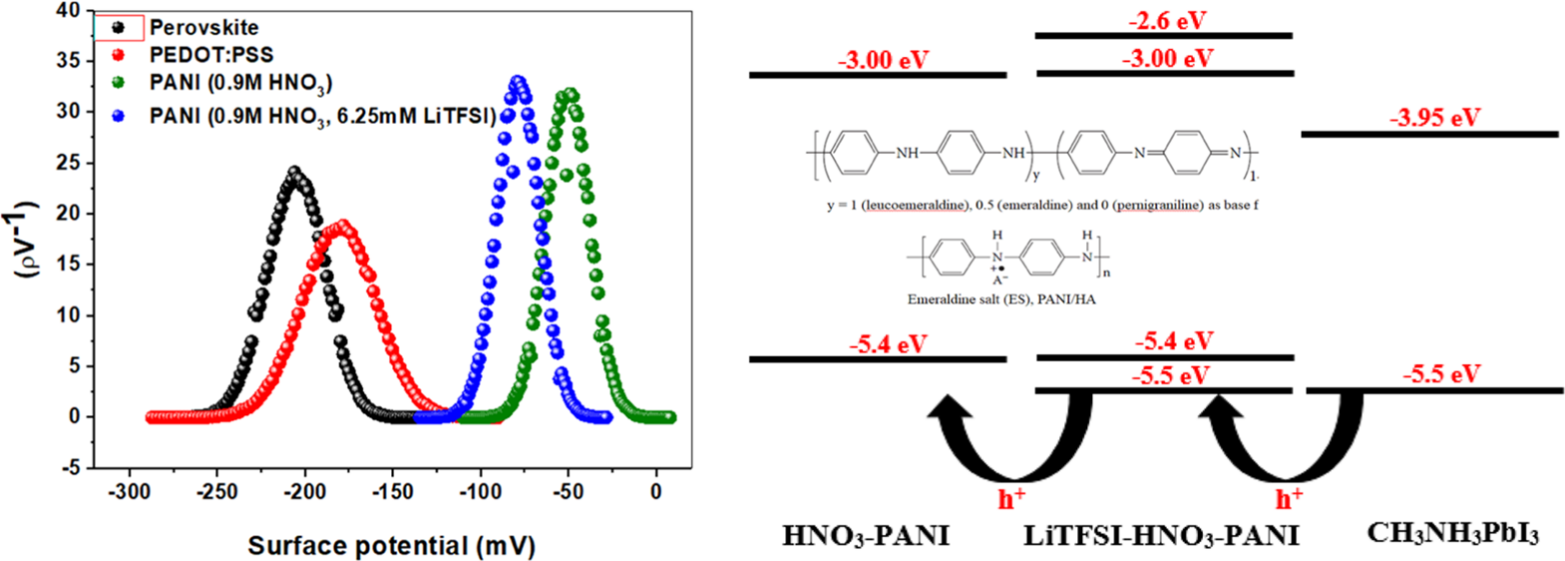

The goal of this work is to substitute the conventional high-cost poly(3,4-ethylenedioxythiophene)-poly(styrenesulfonate) (PEDOT:PSS) in inverted perovskite solar cells (PSCs) with an efficient and conducting polyaniline (PANI) polymer. The reported use of PANI in PSCs involves a chemical synthesis method which is prone to contamination with impurities as it requires several materials for polymerization and adhesion improvement with substrates, contributing to low device efficiencies. This work mitigates this issue using an electrochemical method that is low cost, less time consuming, and capable of producing thin films of PANI with excellent adhesion to substrates. Results demonstrated that the power conversion efficiency of the electrochemically synthesized PANI-based PSC is 16.94% versus 15.11% for the PEDOT:PSS-based device. It was observed that the work function of PANI was lower compared to that of PEDOT:PSS which decreased *V*_OC_ but enhanced hole extraction at the hole transport layer/perovskite interface, thus increasing *J*_SC_. Doping electrolyte solution with lithium bis(trifluoromethanesulfonyl)imide LiTFSI increased the work function of PANI, thus increasing *V*_OC_ from 0.87 to 0.93 V. This method enables simple and scalable synthesis of PANI as a competitive hole transport material to replace rather expensive PEDOT:PSS, thus enabling an important step toward low-cost inverted perovskite photovoltaic devices.

## Introduction

Sunlight is a renewable clean energy source, able to meet world’s electricity demand with only one and a half hour of sunlight. Conversion of sunlight into electricity can be realized using solar cells or photovoltaic (PV) technology and storing in energy-storage devices.^1^ PV devices employing organic–inorganic (hybrid) halide perovskites as the absorber layer have attracted great attention because of the excellent light-harvesting properties of the hybrid perovskites with a tunable band gap, low exciton binding energy (∼45 meV), long charge carrier lifetime, and a long diffusion length.^2−8^ Power conversion efficiencies (PCEs) of the perovskite solar cells (PSCs) have increased from 3.8% in 2009 to 25.6%.^9−20^ The PCE of the nip mesoscopic devices has been demonstrated to be superior to that of the pin or inverted structure. However, the inverted structure is preferred as it enables low-temperature fabrication (<100 °C) compared to 450 °C for the mesoscopic structure. The low-temperature fabrication process can facilitate roll-to-roll manufacturability of the perovskite photovoltaic devices.^21^

Poly(3,4-ethylenedioxythiophene)-poly(styrenesulfonate) (PEDOT:PSS) is the most commonly employed hole transport layer (HTL) between the indium tin oxide (ITO) and perovskite photoactive layer. The PCE of the PSCs using PEDOT:PSS as the HTL has increased remarkably. Because of the different reactivities of polymers with active metals, rhodamine 101 which is a conjugated zwitterion was used as an interlayer at the PCBM/Ag interface to lower the work function of the metal, thus enhance electron collection, and improve PEDOT:PSS-based PSC performance.^22^ Further adding a LiF layer increased *V*_OC_ and FF to achieve a higher PCE of 13.2% compared to 12.1% without LiF.^23^ The transparent PEDOT:PSS doped with methanesulfonic acid achieved a PCE of 11% for the rigid and 8% for the flexible PSCs exhibiting excellent mechanical flexibility in the bending test.^24^ Doping PEDOT:PSS with NaCl improved the PCE from 15.1 to 18.1% as a result of enhancing the electrical conductivity and hole extraction capability of the PEDOT:PSS HTL.^25^ Adding RbCl into PEDOT:PSS enlarged the crystal size, enhanced electrical conductivity and hole transport, and increased the work function of PEDOT:PSS, and thus improved the PCE of the MAPbI_3_ PSC from 13.24 to 16.63% and MA_0.7_FA_0.3_Pb(I_0.9_Br_0.1_)_3_ PSC from 16.13 to 18.30%.^26^ However, various issues have been noticed. Most of all, the large particle size of the PEDOT:PSS induced the degradation of the active layer with formation of defects; in addition, its high cost and low electrical conductivity limit its use as a HTL.^27−31^ For these reasons, it is necessary to develop a new hole transport material (HTM) that provides superior properties along with lower cost than PEDOT:PSS.^21,32−38^

Several inorganic materials were utilized as HTL alternatives to PEDOT:PSS such as CuSCN,^39,40^ CuI,^33,41^ CuO_*x*,_^42,43^ NiO_*x*,_^44,45^ MoO_3,_^46,47^ MoS_2,_^48^ and WO_3_.^42^ Similarly, fully vacuum-processed inverted PSCs using N,N0-di(1-naphthyl)-N,N0-diphenyl-(1,10-biphenyl)-4,40-diamine (NPB) as a HTL combined with the interfacial layer of MoO_3_ were demonstrated to achieve a PCE of 13.7%. Likewise, poly [2,6-(4,4-bis-potassiumbutanylsulfonate-4H-cyclopenta-[2,1-b;3,4-b’]-dithiophene)-alt-4,7-(2,1,3-benzothiadiazole)] (CPE-K) was studied as a HTL for the inverted PSC. A PCE of over 12% with enhanced device stability was demonstrated via enhancing the wetting of the precursor solution of perovskite on the CPE layer, the interfacial hole extraction at the perovskite/ITO interface, and pH-neutral CPE-K solution.^49^ Transparent conducting polymers have high conductivity and good stability which make them promising candidates as alternative HTMs to PEDOT:PSS. Polyaniline is one such candidate as it exhibits attractive attributes such as high conductivity, environmental stability, low cost, easy synthesis, high purity, thin film transparency, and a high degree of processability which are suitable for PSCs.^21^

Synthesis of polyaniline (PANI) involves polymerization of aniline which can be performed chemically, electrochemically, photochemically, or enzyme-catalyzed.^38,50^ Our previous work involved characterization of the inverted PSC based on the chemically synthesized PANI as a HTL and exhibited very poor performance with a PCE of 3.33%.^37^ Chemically prepared polyaniline:poly(styrenesulfonate) (PANI:PSS) has been investigated as a HTM in inverted PSCs which achieved a PCE of up to 11.67%.^51^ PSS-g-PANI achieved a good energy level alignment with the VBM of MAPbI_3_ improving PSC *V*_OC_ and *J*_SC_. PSS-g-PANI mixed with the perfluorinated ionomer (PFI) increased the work function (5.49 eV) and PCE to 12.4% in those with the PSS-g-PANI:PFI compared to 7.8% in the PSC with PEDOT:PSS.^52^ Camphorsulfonic acid-doped PANI (PANI-CSA) as a HTL in the inverted PSCs improved the efficiency and stability of the solar cell achieving up to 15.42%, compared to 14.11% for the device fabricated with PEDOT:PSS.^53^ Large-area PANI films were deposited by the sequential solution polymerization technique for the synthesis of hydrated vanadium pentoxide. The prepared PANI film exhibited excellent electrochromic performance and cycling stability showing great potential in situ deposition of large-area PANI films for the development of optoelectronic modules.^54^ The electrochemical polymerization method by cyclic voltammetry is the preferred method to prepare PANI. The prepared polymer from this method can possess high purity and good adhesion with the hydrophilic substrate compared to the chemically prepared PANI, which is prone to impurities and poor adhesion to the substrate. Electrochemical polymerization facilitates total flexibility with control over various parameters during polymerization such as oxidation state, thickness, adhesion, conductivity, and transparency of PANI via changing the applied voltage, scan rate, polymerization time, monomer and dopant concentration, dopant type, and electrolyte temperature.^21,50,55^

**Figure 1:**
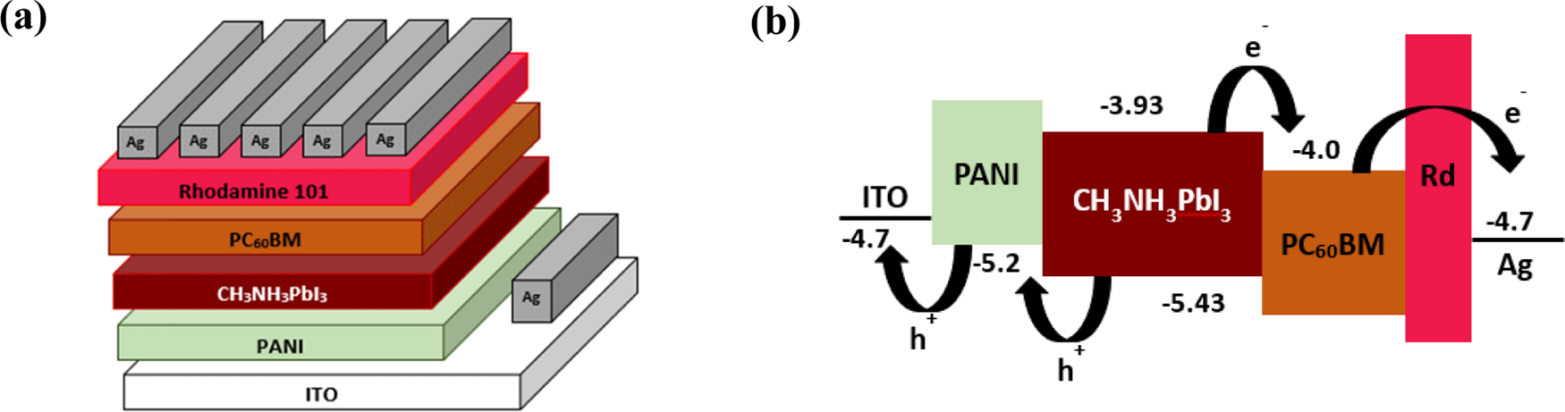
(a) Device structure and (b) energy level diagram of the fabricated PSCs.^21^

This work studies an electrochemically synthesized p-type doped PANI-based HTL via cyclic voltammetry for potential application in PSCs. The synthesized PANI electrode was found to possess high electrical conductivity through doping with nitric acid (HNO_3_). PSCs with the structure shown in Figure 1 were fabricated, and comparative PV studies show that the inverted PSC based on the optimized PANI electrode delivered a higher PCE of 16.94% compared to the device based on the conventional PEDOT:PSS with 15.11%. These results suggest that the PANI prepared using this easy, fast, and low-cost method can be an excellent alternative to the rather expensive PEDOT:PSS to further improve the PV performance and simultaneously reduce the cost of the perovskite PV technology.^21^

## Results and Discussion

### Effect of PANI Annealing Temperature on PSC Performance

Atomic force microscopy (AFM) was used for measuring topography of PANI films annealed at different temperatures. Figure S1, Supporting Information (SI) shows that increasing annealing temperature results in homogeneous and smooth PANI films fully covering the substrate, while the low annealing temperature results in rough PANI and perovskite films with large gaps. X-ray diffraction (XRD) patterns were recorded to understand the effect of the PANI HTL on crystallization of the perovskite films as shown in (Figure S2a, SI). Results confirmed the complete conversion of PbI_2_ to the tetragonal CH_3_NH_3_PbI_3_ perovskite phase with high crystallinity. The highly rough PANI film adsorbs and traps a large amount of perovskite precursor solution resulting in slower interdiffusion of MAI and PbI_2_ into each other, thus slow perovskite crystallization. This results in perovskite films with higher crystallinity when roughness of the underneath PANI film increases.^37^

The conductivities of the electrochemically synthesized PANI on top of ITO substrates and annealed at different temperatures were measured and compared with the conductivity of PEDOT:PSS via using a two-contact electrical conductivity setup (glass/ITO/PANI/Ag) shown in Figure S2b, SI. Figure S2d, SI shows the current–voltage (*I*–*V*) characteristics of the hole-only devices based on the PANI annealed at 60, 100, and 140 °C for 10 min. The following equation was used to measure the conductivity.
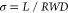
where 1/*R* is the slope of the *I–V* curve while *L*, *W*, and *D* are the length, width, and thickness of the PANI film, respectively. The calculated conductivities were 9.95 × 10^–4^, 8.12 × 10^–4^, and 6.15 × 10^–4^ S/cm for the PANI annealed at 60, 100, and 140 °C, respectively. These results confirm that conductivity of PANI decreases at higher annealing temperatures as a result of degradation of the long polymer chains into shorter ones. Previous reports confirmed that the electrical conductivity of polyaniline degrades with exposure to heated air (100–140 °C) due to corroded macroscopic grain boundaries and degradation caused by changes to the polymer itself, such as a decrease in the dopant concentration, oxidation, and crosslinking.^56^

*J–V* characteristics of the fabricated PSCs Figure S3 and Table S1, SI confirm that the optimum PANI annealing temperature is 100 °C resulting in solar cells with 7.6% PCE which is in good agreement with AFM topography and conductivity measurements.

### Effect of the HNO_3_ Doping Degree of Polyaniline on PSC Performance

AFM topography images Figure S4, SI show that the optimum HNO_3_ concentration which achieves the lowest RMS roughness (3.35 nm) is 0.9 M. *J*–*V* characteristics from Figure S5a,b and Table S2, SI show that the optimum HNO_3_ concentration for doping PANI is 0.9 M resulting in a solar cell with a PCE of 16.19% compared to 15.11% for the PEDOT:PSS-based cell. Transient photocurrent (TPC) data in Figure S5c,d, SI, are in good agreement with PV parameters from *J*–*V* curves (Figure S5a,b, SI). The 0.9 M HNO_3_-based solar cell achieved the lowest charge transport times while carrier lifetime decreased with increasing the doping degree because of the shift of the PANI polaron band to a lower work function.

### Effect of the LiTFSI Doping Degree of PANI-HNO_3_ on PSC Performance

PANI highest occupied molecular orbital (HOMO)–lowest unoccupied molecular orbital (LUMO) measurements were performed before and after doping to study the effect of LiTFSI on the energy level alignment and hole extraction. Results in Figure S6 and Table S3, SI, show the HOMO of HNO_3_-PANI with the value −5.4 eV. Further doping of PANI with LiTFSI resulted in an additional oxidation peak with a HOMO value of −5.5 eV and thus generation of a new energy level with a higher work function. Figure S7 shows a schematic diagram showing energy levels of CH_3_NH_3_PbI_3_, HNO_3_-PANI, and LiTFSI-HNO_3_-PANI. These results expect an increase in the work function of PANI as a result of LiTFSI doping thus expecting improvement in *V*_OC_ of the fabricated device.

AFM topography measurements (Figure S8, SI) confirm that the optimum LiTFSI concentration is 6.25 mM and higher concentration results in a PANI film with higher roughness and thus solar cells with lower *J*_SC_ values.

PSCs made of HNO_3_-PANI achieved 16.19% PCE (Figure S9a,b and Table S4, SI) compared to 16.94% for the solar cell made of LiTFSI-HNO_3_-PANI (6.25 mM LiTFSI) as a result of the improved *J*_SC_ and *V*_OC_. This is attributed to the increased PANI work function and the improved interfacial hole extraction as a result of LiTFSI doping. TPC and transient photovoltage (TPV) results (Figure S9c,d and Table S4, SI) match with *J*_SC_ and *V*_OC_ results from the *J*–*V* characteristics (Figure S9a,b). They confirm that 6.25 mM LiTFSI doping decreased the charge transport time and increased the carrier lifetime which is in good agreement with *V*_OC_ and *J*_SC_ values of the fabricated devices.

**Figure 2:**
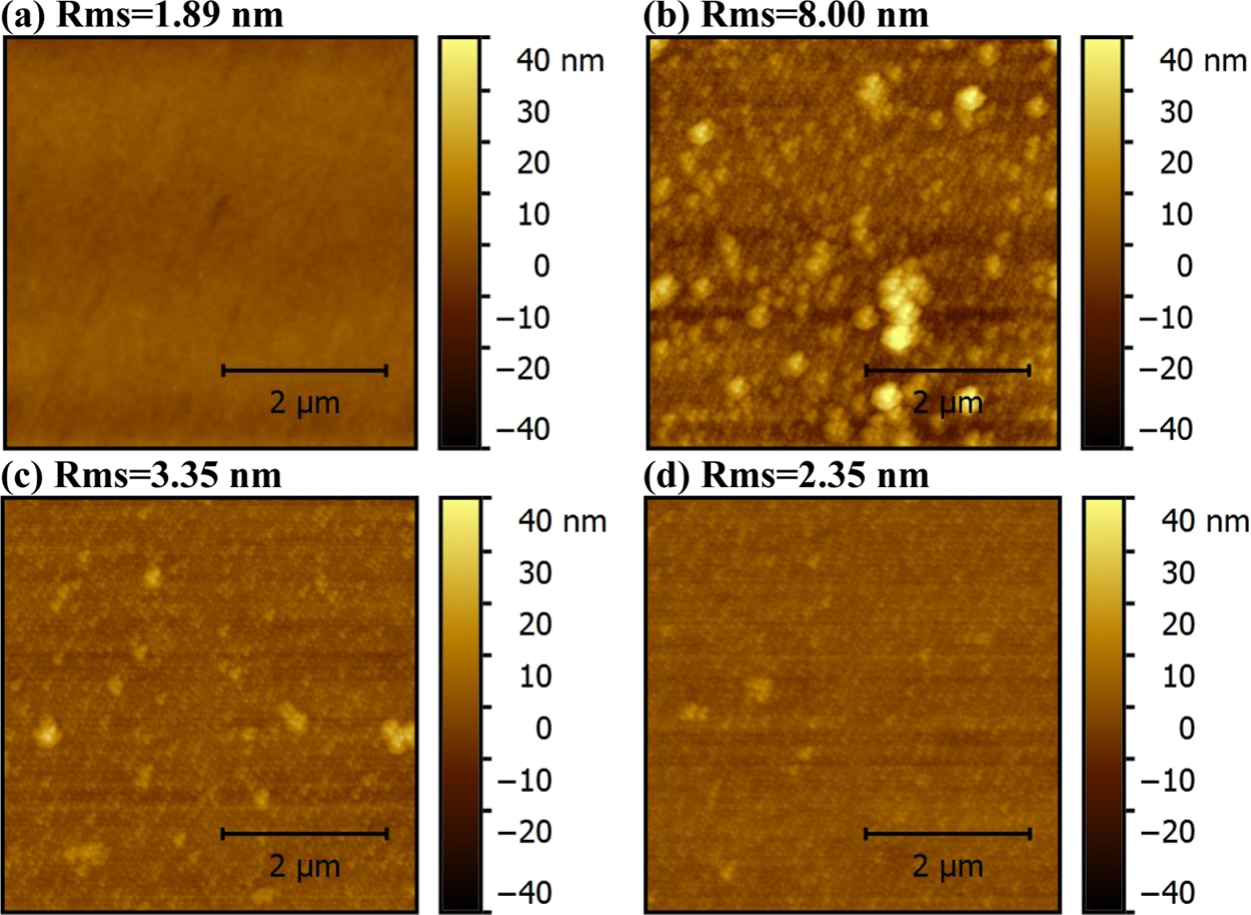
AFM topography images of (a) PEDOT:PSS and electrochemically synthesized HNO_3_-PANI doped with (b) 0.8 M HNO_3_, (c) 0.9 M HNO_3_, and (d) 0.9 M HNO_3_, 6.25 mM LiTFSI.^21^

After optimization of the processing parameters for electrochemically synthesized PANI as shown before, further characterizations were performed for comparing between PEDOT:PSS and PANI as HTLs for the inverted PSC as follows. AFM measurements of PEDOT:PSS and the electrochemically synthesized PANI were carried out, and their topographical images are shown in Figure 2. The PEDOT:PSS film shows a RMS surface roughness of 1.89 nm (Figure 2a), while the PANI film doped with 0.8 M HNO_3_ has a high RMS roughness value of 8.00 nm (Figure 2b). Increasing HNO_3_ concentration to 0.9 M decreases the RMS value to 3.35 nm (Figure 2c), and adding 6.25 mM LiTFSI into the electrolyte solution in addition to 0.9 M HNO_3_ further decreases the RMS value to 2.35 nm (Figure 2d). The decrease in the surface roughness is observed because HNO_3_ and LiTFSI not only act as dopants, but also as catalysts increasing polymerization rate of aniline. This results in full coverage of the substrate surface, thus producing a smoother and more compact PANI film. The lower surface roughness is expected to facilitate charge extraction and collection, thus improving *J*_SC_ for the perovskite device.

**Figure 3:**
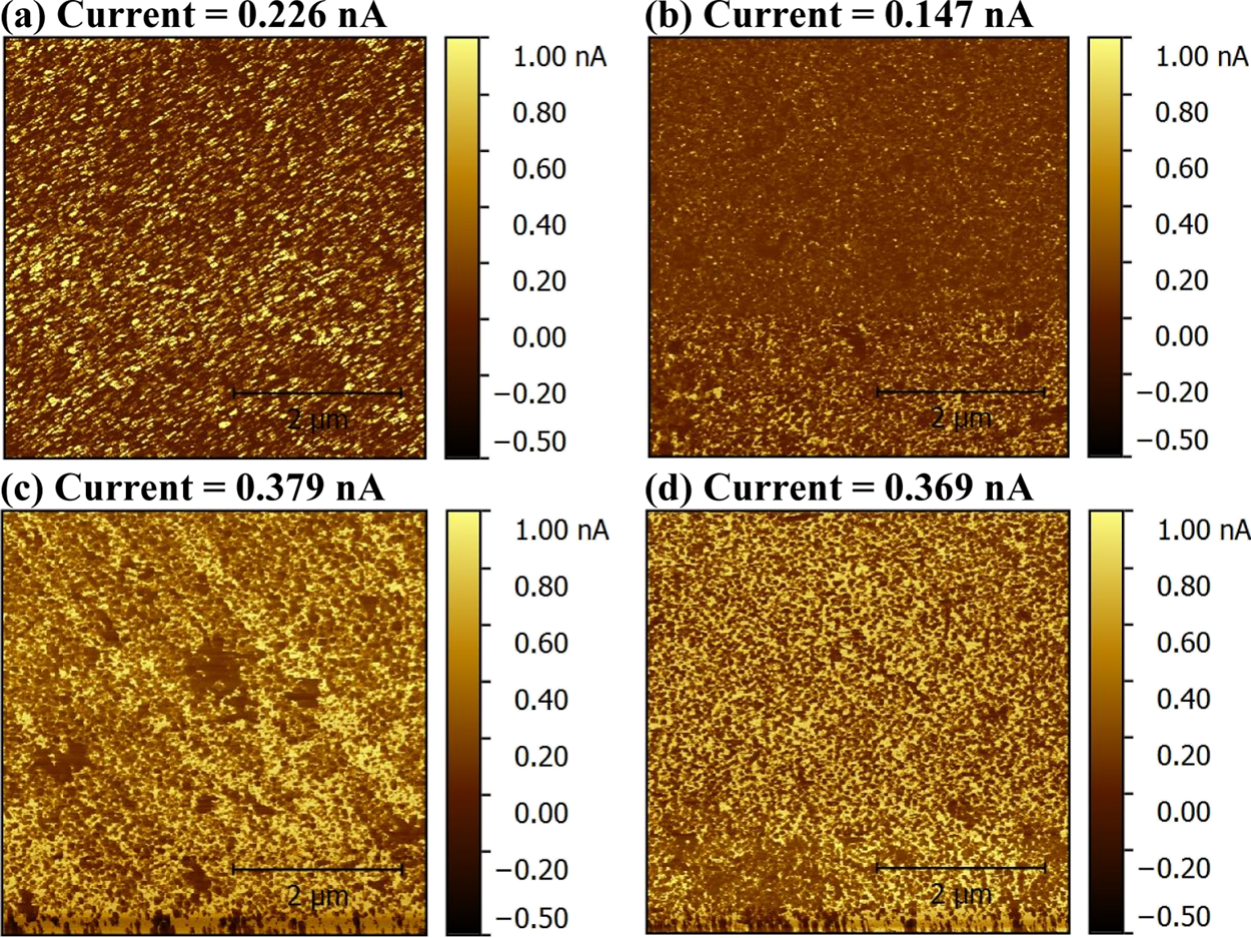
CSAFM images of (a) PEDOT:PSS and electrochemically synthesized HNO_3_-PANI doped with (b) 0.8 M HNO_3_, (c) 0.9 M HNO_3_, and (d) 0.9 M HNO_3_/6.25 mM LiTFSI.^21^

Figure 3 shows CSAFM images of PEDOT:PSS (Figure 3a) and the electrochemically synthesized PANI doped with 0.8 M HNO_3_ (Figure 3b), 0.9 M HNO_3_ (Figure 3c), and 0.9 M HNO_3_/6.25 mM LiTFSI (Figure 3d) exhibiting surface current values of 0.226, 0.147, 0.379, and 0.369 nA, respectively. This shows that the surface current of electrochemically synthesized PANI increases with increasing the HNO_3_ concentration in the electrolyte solution. To understand the change in surface current as a result of doping, conductivities of the electrochemically synthesized PANI on top of ITO substrates were measured before and after optimization of HNO_3_ and LiTFSI dopants and compared with the conductivity of PEDOT:PSS using a two-contact electrical conductivity setup (glass/ITO/PANI/Ag) and (Ag/PEDOT:PSS/Ag) as shown in Figure S2b,c, SI respectively. Figure S2e shows the current–voltage (*I*–*V*) characteristics of the hole-only devices based on the PANI doped with 0.8 M HNO_3_, 0.9 M HNO_3_, and 0.9 M HNO_3_ + 6.25 mM LiTFSI and annealed at 100 °C for 10 min. Figure S2f, SI, shows the *I*–*V* characteristics of the hole-only device based on PEDOT:PSS annealed at 140 °C for 10 min. Conductivity results match with surface current measurements at which increasing HNO_3_ concentration from 0.8 to 0.9 M in the electrolyte solution enhanced conductivity significantly from 8.12 × 10^–4^ to 1.05 × 10^–3^ S/cm, respectively. Further adding 6.25 mM LiTFSI slightly decreased the conductivity of PANI to 8.69 × 10^–4^ S/cm compared to 8.43 × 10^–4^ S/cm for PEDOT:PSS. This is because polyaniline conductivity depends on its oxidation state and protonation degree. When electrochemical polymerization of the aniline monomer into PANI is performed, aniline is oxidized into an anilinium cation radical at the N position which delocalizes by conjugation into O- and P- positions followed by coupling between radicals at N- and P- positions. Therefore, the N atom is involved in the polymer chain and its oxidation state greatly affects PANI’s conductivity. If all N atoms are oxidized into imine groups, PANI is called pernigraniline base (violet) and if all N atoms are reduced into amine groups, PANI is called leucoemeraldine base (yellow). If PANI is half oxidized, it is called emeraldine base, which is the only form that could be conductive via protonation of imine groups giving the conducting emeraldine salt (green). This could be achieved by doping the electrolyte solution with a strong acid such as HNO_3_. Decreasing the HNO_3_ concentration leads to some unprotonated imine groups, thus decreasing conductivity. As a result, the PANI film doped with 0.9 M HNO_3_ has higher conductivity and surface current than the film doped with 0.8 M HNO_3_. Adding LiTFSI into the electrolyte solution can protonate some amine groups and thus decrease the conductivity and surface current of PANI.^21^

**Figure 4:**
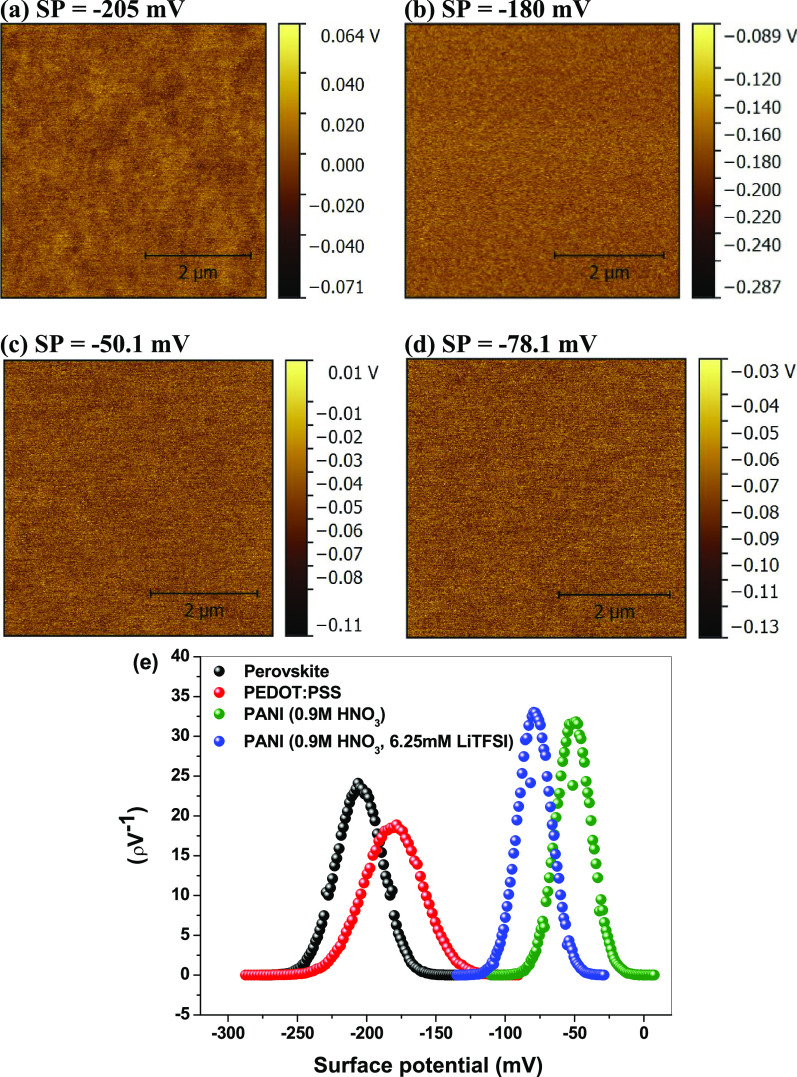
KPFM images of (a) CH_3_NH_3_PbI_3_ perovskite, (b) PEDOT:PSS, (c) PANI doped with 0.9 M HNO_3_, and (d) PANI doped with 0.9 M HNO_3_ and 6.25 mM LiTFSI, and (e) their surface potential distribution.^21^

Figure 4 shows the KPFM images of the perovskite film (Figure 4a), PEDOT:PSS film (Figure 4b), and the electrochemically synthesized PANI film doped with 0.9 M HNO_3_ (Figure 4c) and 0.9 M HNO_3_/6.25 mM LiTFSI (Figure 4d) and their surface potential distribution (Figure 4e) acquired from the KPFM images. Results show that the PEDOT:PSS has a higher work function than the PANI films suggesting higher *V*_OC_ for the PEDOT:PSS-based cell than the PANI-based cells. The PANI doped with LiTFSI has a higher work function (surface potential = −78.1 mV) compared to the PANI without LiTFSI (surface potential = −50.1 mV) suggesting that adding LiTFSI can result in enhancement of *V*_OC_. This might be attributed to that LiTFSI increased the work function of PANI due to the generation of new HOMO energy as shown by HOMO–LUMO measurements (Figure S6, SI).^21^

**Figure 5:**
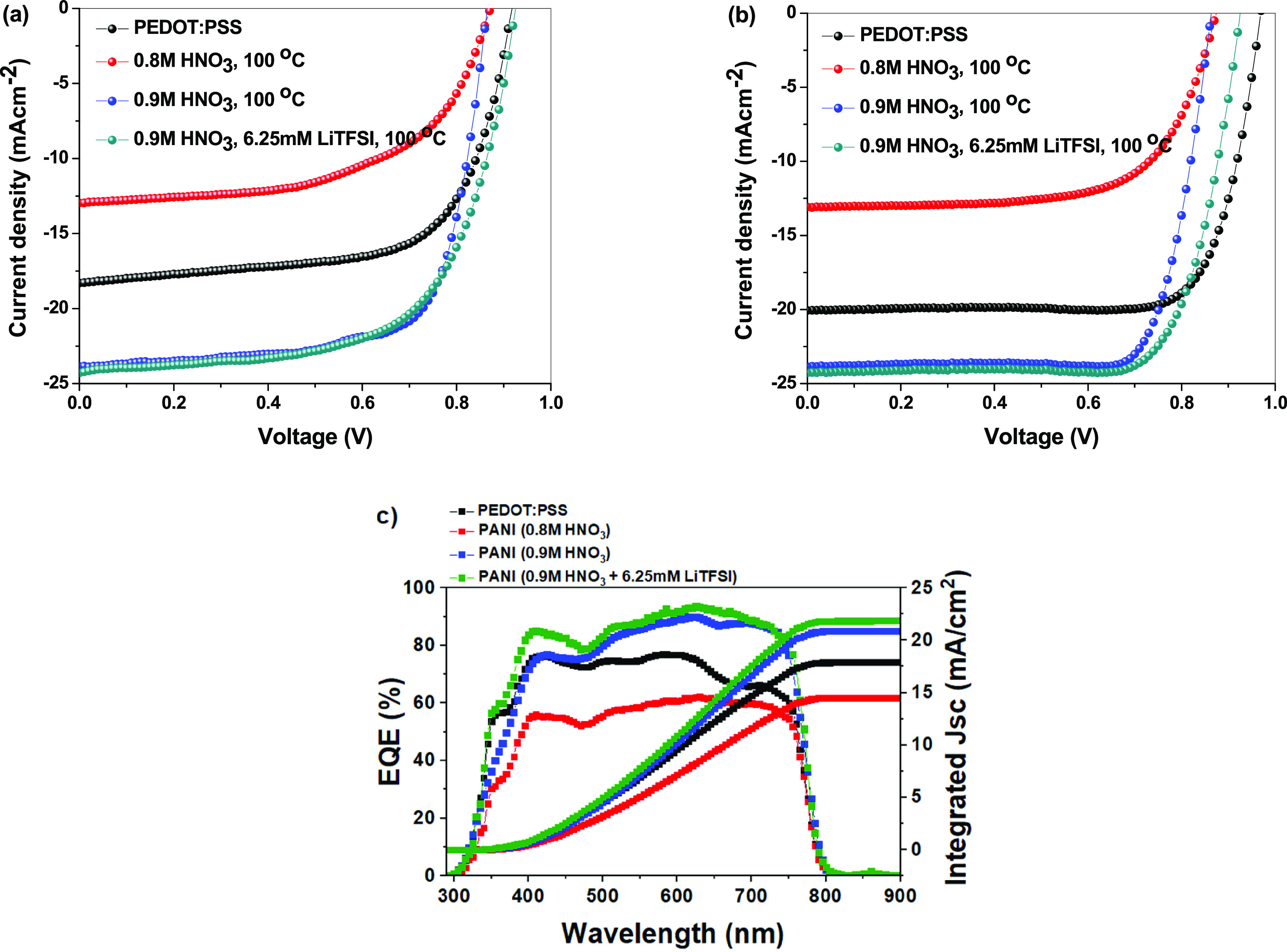
(a) Forward and (b) reverse *J*–*V* characteristics of PSCs based on PEDOT:PSS and electrochemically synthesized PANI films as HTMs. (c) IPCE spectra and integrated current density of PSCs based on PEDOT:PSS and the optimized electrochemically synthesized PANI films as HTMs.^21^

Figure 5a,b show forward and reverse J–V curves of PSCs based on PANI doped with different concentrations of HNO_3_ and LiTFSI as HTMs compared to the PEDOT:PSS-based cell, and Table 1 summarizes the PV performance parameters of the fabricated devices. Results show that the device made of PANI doped with 0.8 M HNO_3_ achieved a PCE of 7.61%. Increasing the doping degree to 0.9 M HNO_3_ enhances PCE of the PANI-based devices to 16.19%, which is higher compared to that of the PEDOT:PSS-based cell with a PCE of 15.11%. The enhanced efficiency is attributed to the improved *J*_SC_ and FF as a result of increasing doping degree, which is in good agreement with the AFM, conductivity, and CSAFM observations. This is because the protonation degree of imine groups increases with increasing the doping degree, leading to an increase in surface current and conductivity. In addition, the decrease in PANI film roughness can also contribute to enhancement in FF and *J*_SC_.^21^

**Table 1:** PV Performance Parameters of Perovskite Solar Cells Based on PEDOT:PSS and Electrochemically Synthesized PANI Films as HTMs^21^

HTM	scan direction	*J*_SC_ (mAcm^–2^)	*V*_OC_(V)	FF	PCE (%)	IPCE (%)	Integrated *J*_SC_ from IPCE (mAcm^–2^)
PEDOT:PSS	forward	18.31	0.92	0.65	11.02	76.97	17.93
	reverse	20.08	0.97	0.78	15.11		
PANI (0.8 M HNO_3_)	forward	12.97	0.87	0.57	6.39	62.23	14.5
	reverse	13.11	0.88	0.66	7.60		
PANI (0.9 M HNO_3_)	forward	23.88	0.87	0.7	14.54	89.90	20.88
	reverse	23.86	0.87	0.78	16.19		
PANI (0.9 M HNO_3_, 6.25 mM LiTFSI)	forward	24.28	0.93	0.63	14.23	93.56	21.87
	reverse	24.28	0.93	0.75	16.94		

Adding 6.25 mM LiTFSI into the electrolyte solution results in further enhancement in solar cell efficiency up to 16.94% which is attributed to the improved *V*_OC_ and *J*_SC_. The enhanced *V*_OC_ is in good agreement with the KFM results (Figure 4), which confirmed that doping PANI with LiTFSI increased its work function, thus increasing *V*_OC_. LiTFSI enhanced the *J*_SC_; however, CSAFM images showed that it decreased surface current of PANI as a result of doping amine groups. The enhanced *J*_SC_ can be attributed to that LiTFSI generated a new hole transporting energy level between the HOMO of perovskite and HOMO of PANI, thus facilitating charge extraction and collection at the interface. This confirms that further doping of PANI with LiTFSI can introduce some amine groups resulting in little decrease in surface current of the PANI film. At the same time, doping of PANI with LiTFSI resulted in the generation of additional energy levels with work functions higher than PANI and lower than perovskite and thus enhanced the interfacial hole extraction at the HTL/perovskite interface in the full device. The increase in *J*_SC_ of the solar cell as a result of the enhancement of the PANI/perovskite interfacial charge extraction is greater than the decrease in *J*_SC_ as a result of the reduction of the PANI film surface current after doping with LiTFSI.^21^

Hysteresis, that is, discrepancy that exists between forward and reverse voltage sweeping during *I*–*V* measurement was found in all the cells. This is because PV parameters greatly depend on the voltage range, scan rate, and the direction of the applied bias. In PSCs, hysteresis increases in case of poor contact quality (surface recombination), a short diffusion length of the charge carriers, ion migration and polarization, charge trapping/detrapping process, and material and interface defects. The most common reason for hysteresis in perovskites is mainly ion migration due to excess ions as interstitial defects. This is because perovskites have ionic characteristics and can be segregated into positively charged and negatively charged ionic species leading to ion migration. The accumulation of the excess ions can facilitate the polarization of the perovskite leading to hysteresis. Results show a significant decrease in *I–V* curve hysteresis of the PSC based on electrochemically synthesized PANI after increasing HNO_3_ dopant concentration from 0.8 to 0.9 M. This is in good matching with conductivity and surface current measurements and could be attributed to the enhancement in PANI conductivity and surface current as a result of optimization of the protonation degree. The enhancement in the electronic conductivity of PANI suppresses the accumulation and recombination of charge carriers in the bulk and at the interfaces and thus mitigates hysteresis.^21,57^

The IPCE spectra (Figure 5c) are in good agreement with the *J*_SC_ values obtained from the J–V characteristics. As shown in Table 1, IPCE of the 0.8 M HNO_3_-PANI-based solar cell is 62.23% compared to 76.97% for the PEDOT:PSS-based device. Increasing the doping degree of PANI to 0.9 M HNO_3_ increased IPCE to 89.90% as a result of increasing protonation of imine groups thus achieving higher conductivity. Also, doping with 6.25 mM LiTFSI improved IPCE up to 93.56% because of the enhanced charge extraction as a result of generation of a new HOMO energy level between those of perovskite and PANI. These results perfectly match conductivity, surface current, HOMO–LUMO, and surface potential measurements which confirm that increasing HNO_3_ concentration in the electrolyte solution during electrochemical synthesis of PANI resulted in higher surface current as a result of protonation of imine groups in the polymer chain. While HOMO–LUMO and KPFM measurements confirm that doping PANI with LiTFSI resulted in the generation of a new energy level with a higher work function compared to that of PANI, which enhanced charge extraction at the PANI/perovskite interface.^21^

The integrated *J*_SC_ values of the prepared devices were calculated from ICPE and are listed in Table 1; they exhibit little lower values compared to the *J*_SC_ from the *J*–*V* curves. This is a common phenomenon in thin-film PV devices due to the barrier for the photocurrent, which is large under low light intensity or monochromatic illumination but becomes lowered by photodoping of the buffer at AM1.5 illumination. In addition, EQE measurement involves irradiation of a limited area in the device which may have small shunt resistance, while the nonilluminated part in the cell has high shunt resistance in parallel with the EQE amplifier resistance (act as shunting load). Thus, the current from the active solar cell is drained throughout the shunting load. Sometimes, the *J*_SC_ from the *J–V* curve is lower than that from IPCE; this is because a small thermionic emission current can pass the barrier during the EQE measurement, while the high current density under AM1.5 illumination cannot pass the barrier.

Figure S10a shows a histogram of the PCE from 20 individual PSCs based on PEDOT:PSS and the electrochemically synthesized PANI before and after doping with LiTFSI. The PCE histogram from 20 samples of each HTL (Figure S10a) demonstrates the better reproducibility for PEDOT:PSS (SD = ±0.562%) compared to PANI (SD = ±0.906%). Doping PANI with LiTFSI results in better and more reproducible solar cell performance with SD = ±0.566%. The average PCE for the PANI-based PSC before and after LiTFSI is 14.54 and 15.89%, respectively, compared to 14.37% for the PEDOT:PSS-based PSC. The enhancement in reproducibility of the PSC based on PANI after LiTFSI could be attributed to the enhanced carrier life time and *V*_OC_ which plays an important role in suppression of charge carrier recombination.

Figure S10b indicates the stability of devices via showing the normalized PCE evolution over time of unencapsulated PSCs based on PEDOT:PSS and the optimized electrochemically synthesized PANI HTLs under the ambient condition (23 ± 1 °C and 50 ± 5% humidity). The PEDOT:PSS-based device degrades completely after 600 h exposure to air and humidity at room temperature, because the large particle size of the PEDOT:PSS induced the degradation of the active layer with formation of defects suppressing its stability, while the PANI-based device is still working after around 800 h. These results confirm that the PANI HTL improves the device stability compared to PEDOT:PSS. This is because polyanilines are very stable in air and the conductivity stability of PANI is enhanced in the presence of humid air. In addition, PANI is highly stable thermally (up to 450 °C for the undoped emeraldine base), and doping may decrease its thermal stability based on the nature of counter anions (acid dopant).^58^ Previous reports confirmed that PANI is also highly stable in air and acidic water.^59^

**Figure 6:**
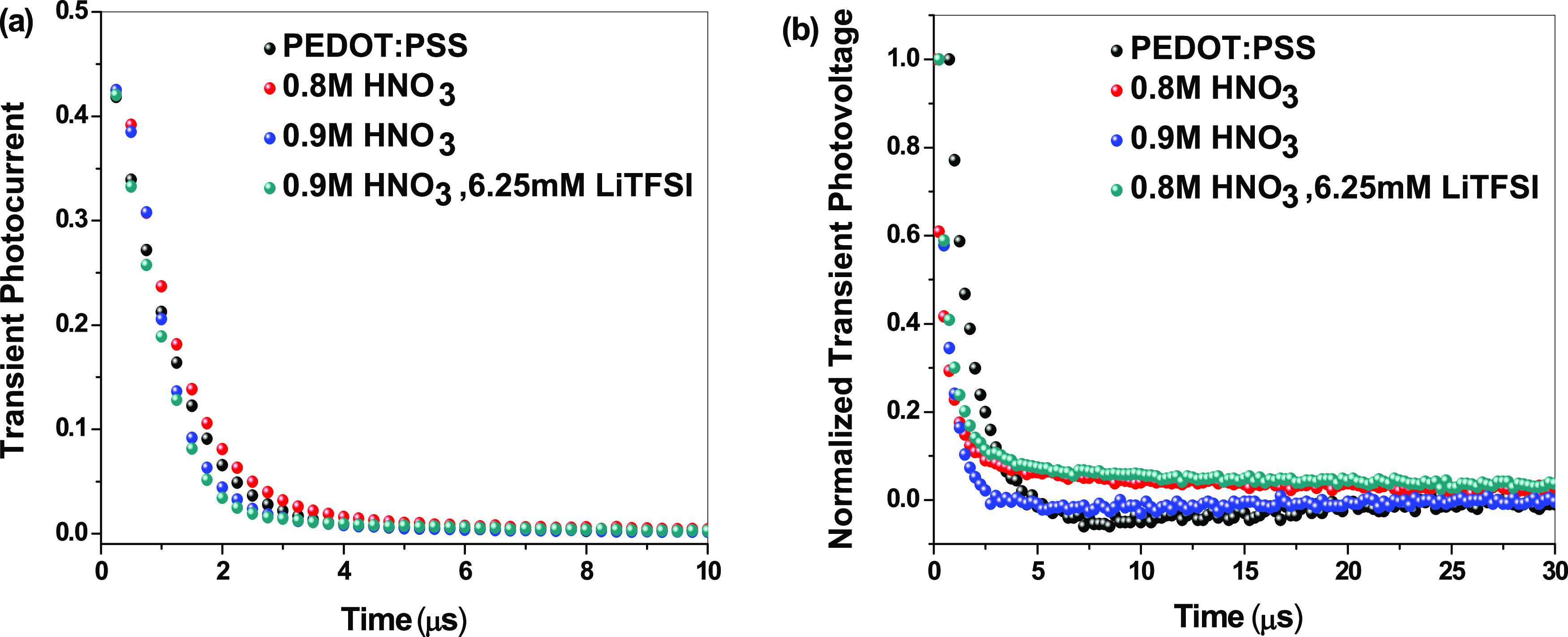
(a) Transient photocurrent and (b) normalized transient photovoltage of inverted PSCs based on PEDOT:PSS and electrochemically synthesized PANI films as HTMs.^21^

Figure 6 shows TPC and TPV measurements of PSCs based on PEDOT:PSS and the different PANI films as HTMs, and Table 2 shows the obtained values of charge carrier transport time and lifetime. A charge transport time of 1.08, 0.86, and 0.81 μs was obtained for the cells fabricated with PANI HTMs doped with 0.8 M HNO_3_, 0.9 M HNO_3_, and 0.9 M HNO_3_/6.25 mM LiTFSI, respectively, compared to the cell based on pristine PEDOT:PSS with 0.99 μs. These results match with the observations made in the J–V characteristics as the lower transport time values further confirm the positive effect of the doping degree and dopant type on charge extraction, thus enhancing *J*_SC_ values of the inverted PSCs.^21^

**Table 2:** Fitted Charge Carrier Transport Time and Lifetime for Perovskite Solar Cells Based on PEDOT:PSS and Electrochemically Synthesized PANI Films as HTMs^21^

HTM	charge carrier transport time (μs)	Charge carrier lifetime (μs)
PEDOT:PSS	0.99	1.02
PANI (0.8 M HNO_3_)	1.08	0.72
PANI (0.9 M HNO_3_)	0.86	0.53
PANI (0.9 M HNO_3_, 6.25 mM LiTFSI)	0.81	0.75

Similarly, the TPV results are in good agreement with the *V*_OC_ values obtained from the *J–V* characteristics. Charge carrier lifetime of the devices based on PEDOT:PSS is the longest with 1.02 μs compared to the electrochemically synthesized PANI films. Increasing the doping degree from 0.8 to 0.9 M HNO_3_ decreased carrier lifetime from 0.72 to 0.53 μs. Further doping HNO_3_-PANI with LiTFSI results in an increase in carrier lifetime to 0.75 μs thus improving *V*_OC_ from 0.87 to 0.93 V. These enhancements in the charge carrier dynamics parameters match with the KPFM measurements (Figure 4) as LiTFSI generates a new hole transporting energy level between the HOMO of perovskite and the HOMO of PANI and increasing the work function of the PANI, thus improving *V*_OC_. Also, the new energy level facilitates enhancement in charge extraction and collection in the HTL resulting in shorter transport time and improved *J*_SC_.

## Conclusions

A new approach aimed toward commercialization of PSCs has been implemented via substituting conventional high-cost PEDOT:PSS by facile synthesis of polyaniline electrochemically. This can solve the issue of contamination in the chemical synthesis method as it requires several materials for polymerization and adhesion improvement with substrates. The PANI thin film was synthesized by a simple and scalable electrochemical method and implemented as a HTM for the inverted PSCs. A competitive PCE of 16.94% was achieved for the electrochemically prepared PANI-based inverted PSC compared to the conventional PEDOT:PSS with 15.11%. The PANI-based PSC achieved better *J*_SC_ and lower *V*_OC_ and FF compared to the PEDOT:PSS-based device. This can be attributed to the work function of PANI being lower compared to that of PEDOT:PSS which decreased *V*_OC_ but enhanced hole extraction at the HTM/perovskite interface, thus increasing *J*_SC_. Doping electrolyte solution with LiTFSI increased the work function of PANI, thus increasing *V*_OC_ from 0.87 to 0.93 V. This work presents a facile route to produce a cheaper alternative to PEDOT:PSS, potentially contributing toward low-cost PSCs.^21^

## Supporting Information Available

Experimental work including materials, PANI synthesis procedure, device fabrication, and characterization, optimization of the parameters of PANI electrochemical synthesis, AFM topography images of PANI films and perovskite deposited on top of it, XRD of perovskite on top of PANI annealed at different temperatures, conductivity of PANI compared to PEDOT:PSS, *J–V* curves of optimizing the annealing temperature and doping degree of PANI, transient measurements of devices based on PEDOT:PSS and PANI, HOMO–LUMO measurement of PANI before and after doping and a schematic diagram of energy level alignment, a histogram showing reproducibility of PEDOT:PSS- and PANI-based devices, and a curve showing the stability of PSC devices (PDF)
