# Supplementary material for: Electrochemically Prepared Polyaniline as an Alternative to Poly(3,4-ethylenedioxythiophene)-poly(styrenesulfonate) for Inverted Perovskite Solar Cells
Source: ACS Appl Energy Mater. 2022 Jul 20;5(8):9351–60. doi: 10.1021/acsaem.2c00621 (PMC9400027; doi:10.1021/acsaem.2c00621)
Supplement: Supplementary file 1 — ae2c00621_si_001.pdf [file ae2c00621_si_001.pdf]

Supporting Information

Electrochemically Prepared Polyaniline as an Alternative to PEDOT:PSS for Inverted Perovskite Solar Cells

Sally Mabrouk[1], Ashim Gurung[2], Behzad Bahrami[2], Abiral Baniya[1], Raja Sekhar Bobba[1], Fan Wu[3], Rajesh Pathak[2], Quinn Qiao[1,*]

[1]Mechanical and Aerospace Engineering, Syracuse University, Syracuse, NY 13244

[2]Center for Advanced Photovoltaics, Department of Electrical Engineering and Computer Science, South Dakota State University, Brookings, SD 57007

3Key Lab of Optoelectronic Materials and Devices, School of Science, Huzhou University, Huzhou, 313000, China

*Corresponding author: Email: quqiao@syr.edu

Experimental Work

1. Materials

Methylammonium iodide (CH_3NH_3I) was purchased from Dyesol. Lead iodide (PbI_2) was purchased from Acros Organics. Indium tin oxide (ITO) coated glass substrates were purchased from Hartford Glass Company. Aniline was obtained from Acros organics while nitric acid was ordered from Fischer scientific. [6,6]-Phenyl-C61-butyric acid methyl ester ($PC_{60}BM$) was obtained from Nano-C. PEDOT:PSS was purchased from Heraeus grade Clevios P VP AI 4083 [1].

2. Device fabrication

Perovskite solar cells were fabricated on ITO coated substrates (1.5 cm × 1.0 cm). ITO substrates were cleaned by sonication in soap water, DI-water, acetone and isopropanol for 25 min each, then treated with UV-ozone plasma for 3-5 min. A thin layer of PANI was synthesized electrochemically by cyclic voltammetry on the cleaned ITO substrates. Electro-polymerization of aniline was carried out using these cleaned ITO substrates as a working electrode with a total exposure area of 1 cm^2. Pt wire was used as an auxiliary (counter) electrode and a Ag/AgCl as a reference electrode in an aqueous solution containing 0.5M aniline monomer and different concentrations of HNO_3 (0.8M, 0.9M, 1.0M) and Lithium bis(trifluoromethanesulfonyl)imide (LiTFSI) salt (0 mM, 6.25 mM, 12.5mM, 50.0 mM) as dopants via cyclic voltammetry at a

potential interval range from (-0.2 to 1.1V), sweep rate of 25 mVs^{-1} for 1 cycle using a computer controlled potentiostat. After electro-polymerization, PANI film was washed with distilled water followed by annealing at different temperatures (60, 100, 140 ^oC) for about 10 min. Alternatively, aqueous PEDOT:PSS solution was spin coated at 4500 rpm for 1.0 min on cleaned ITO substrate and annealed at 140 ^oC for 10 min. Perovskite precursor solution consisting of 581mg PbI_2 and 209mg CH_3NH_3I was dissolved in 1 ml solvent consisting of DMSO and ɤ-butyrolactone with 3:7 volume ratio. The prepared solution was stirred on a hotplate inside the glovebox for about 2 hr at 70 ^oC. The perovskite solution was spin coated on top of PANI and PEDOT:PSS films at 750 rpm for 20 sec, followed by spinning at 4000 rpm for 1 min, and toluene was used as anti-solvent. The films were then annealed at 80 ^oC for 10 min. $PC_{60}BM$ solution with a concentration of 20mg/ml in chlorobenzene was spin coated on the top of perovskite layer at 2000 rpm for 40 sec followed by annealing at 80 ^oC for 5 min. Rhodamine solution with 0.5 mg/ml in isopropanol was spin coated on the top of $PC_{60}BM$ layer at 4000 rpm for 40 sec inside glove box. Finally, 100 nm of silver was evaporated as a top electrode [1].

3. *Characterization*

Crystallization and phase identification of the perovskite thin film on the top of PANI film was recorded from RigakuSmartlab X-ray diffractometer (XRD). Topography and kelvin probe force microscopy (KPFM) of thin PANI films were measured using atomic force microscopy method in tapping mode which uses a conducting tip as a Kelvin probe to measure surface potential. An Agilent SPM 5500 atomic force microscope equipped with a MAC III controller (comprising three lock-in amplifiers) used to map surface potential of PANI. A Budget Sensors Multi 75- EG tip having a platinum/iridium conductive coating was used. Current sensing atomic force microscopy (CS-AFM) was measured in contact mode using an Agilent 5500 scanning probe microscope equipped with a MAC-III controller and Pt/Ir coated Si tip. The conducting probe makes contact with the scanned film and measures the current variation across the surface with fixed bias. Hole carriers were injected from a conducting Pt/Ir coated AFM tip into PANI film and collected at the grounded ITO electrode. The current was measured using an in-built preamplifier with 1 nAV^{-1} sensitivity. The images were taken at 1 V bias to avoid tip-induced local oxidation/reduction and impurities on the surface of the film. Current density-voltage curves of cells were measured using Agilent 4155C under AM 1.5 illumination. A solar simulator (Xenon lamp, Newport) was used as a light source with illumination of ~100 mW/cm^2. Light intensity

was calibrated using a National Renewable Energy Laboratory (NREL) Si solar cell (S1133 14-01). The scan rate was $0.5Vs^{-1}$, and the scan was performed in both forward and reverse directions. The external quantum efficiency (EQE) was measured using a monochromator (Newport) attached with a Xenon lamp (Newport) as light source. The light was focused on the solar cell using two focusing lenses. NREL calibrated photodiodes were used as a reference for EQE measurement. Transient measurements were performed using OBB's Model OL-4300 nitrogen laser (crisp pulse at 337 nm) to pump the model 1011 dye laser to generate short pulse, which acted as an excitation source with a pulse duration < 1 ns and a repetition rate ~ 4 Hz [1].

Effect of PANI annealing temperature on PSC performance

Morphology of PANI and PANI/$CH_3NH_3PbI_3$ films annealed at different temperatures were measured using atomic force microscopy (AFM). All AFM samples were prepared in the same processing conditions as the fabricated solar cells. AFM topography images of PANI films (Fig. S1a, S1b, S1c, supporting information) show very high agglomeration for PANI particles when annealed at low temperature 60 ^{o}C (Fig. S1a, SI) resulting in high roughness and PANI film doesn't fully cover the ITO surface. This low temperature is not enough for completely drying the PANI film and the water residues may degrade the perovskite film deposited on top of it. Agglomeration decreases by increasing annealing temperature, thus PANI particles become more distributed, decreasing roughness of PANI film, and substrate surface become completely covered with the HTM. Roughness of annealed PANI film is in the order of 60 ^{o}C > 100 ^{o}C > 140 ^{o}C with the root mean square (rms) roughness values order of 38.7 nm > 16.1 nm > 7.72 nm.

AFM image of the perovskite film prepared on top of PANI film annealed at 60 ^{o}C (Fig. S1d, supporting information (SI)) shows large gaps confirming that perovskite crystals are highly agglomerated resulting in very rough perovskite film as a consequence of the highly rough surface of the underneath PANI layer. Agglomeration and gaps of perovskite film decrease gradually by increasing PANI annealing temperature resulting in more compact and denser perovskite film with lower roughness. Perovskite films prepared on top of PANI film annealed at 100 ^{o}C and 140 ^{o}C have rms roughness values of 17.7 nm (Fig. S1e, SI) and 15 nm (Fig. S1f, SI), respectively which are lower compared to the roughness of the perovskite film on top of PANI annealed at 60 ^{o}C with 22.5 nm (Fig. S1d, SI) [1].

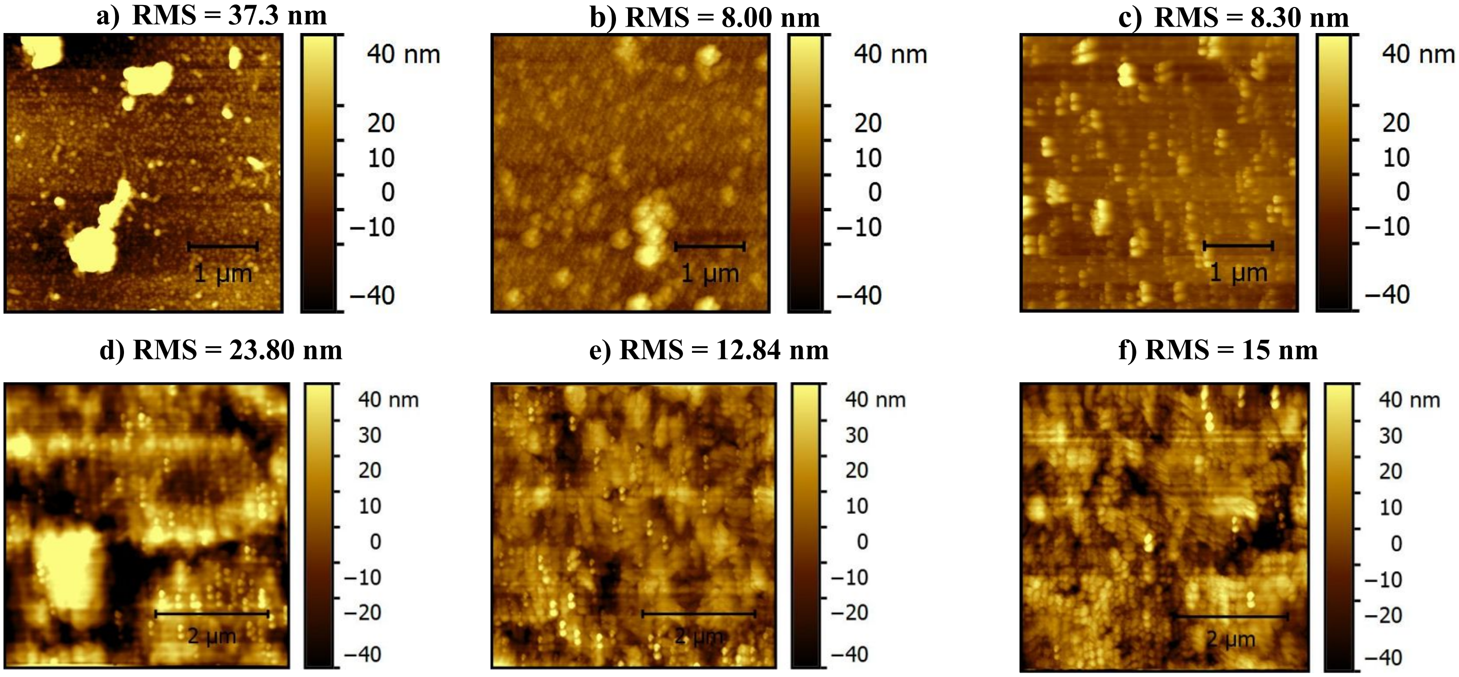

Fig. S1. AFM topography of PANI films (3×3 μm) annealed at different temperatures (a) 60 ^{o}C, (b) 100 ^{o}C, (c) 140 ^{o}C and $CH_3NH_3PbI_3$ films (5×5 μm) on top of the PANI films annealed at different temperatures (d) 60 ^{o}C, (e) 100 ^{o}C, (f) 140 ^{o}C [1].

XRD patterns were recorded to understand the effect of PANI hole transport layer on crystallization of the perovskite films as shown in(Fig. S2a, SI). XRD Bragg peaks at 14.06°, 28.41°, 31.85°, and 43.19° represent (110), (220), (310), and (330) crystal planes of the tetragonal $CH_3NH_3PbI_3$ perovskite, respectively. This indicates the high crystallinity of perovskite films coated on top of PANI film. The peak at 12.52° which is assigned to (001) of PbI_2 is not present in the XRD data which indicates complete conversion of PbI_2 to perovskite. Crystallinity of perovskite prepared on top of PANI follows the order of annealed at 60 ^{o}C > 100 ^{o}C > 140 ^{o}C, this is in good agreement with the surface roughness of PANI, which increases with decreased annealing temperature. The highly rough PANI will adsorb and trap large amount of perovskite precursor solution which leads to the slower inter-diffusion of MAI and PbI_2 into each

other, thus slow perovskite crystallization. This results in perovskite film with higher crystallinity when roughness of the underneath PANI film increases [1-2].

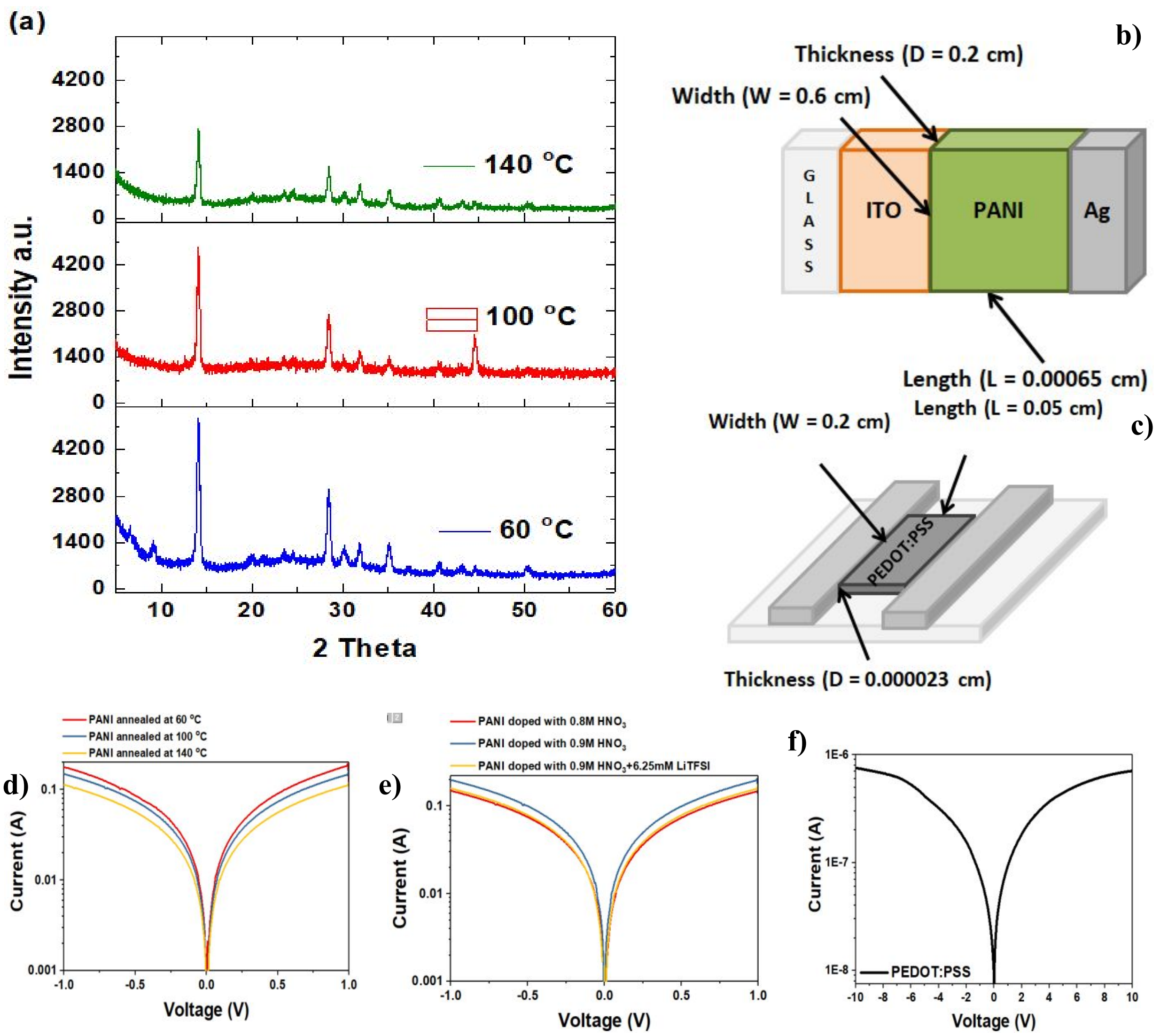

Fig. S2. a) XRD spectrum of $CH_3NH_3PbI_3$ films on top of the PANI films annealed at different temperatures (60 °C, 100 °C, 140 °C) [1]. Two-contact electrical conductivity set-up b) (glass/ITO/PANI/Ag) and c) (Ag/PEDOT:PSS/Ag). d) IV curves of hole-only devices based on the PANI doped with 0.8M HNO_3 and annealed at 60 °C, 100 °C, and 140 °C for 10 min. e) IV curves of hole-only devices based on based on the PANI doped with 0.8M HNO_3, 0.9M HNO_3,

and 0.9M HNO_3 + 6.25mM LiTFSI and annealed at 100 °C for 10 min. f) IV curve of hole-only device based on PEDOT:PSS annealed at 140 °C for 10 min.

Figure S3 shows J-V characteristics of the perovskite solar cells using the electrochemically synthesized PANI annealed at different temperatures and table S1 summarizes the photovoltaic performance parameters of the fabricated devices. Results show that the PANI based devices dried at 60 °C achieved power conversion efficiency (PCE) of up to 5.81%. Increasing annealing temperature to 100 °C enhances PCE to 7.60%, while beyond 100 °C, efficiency start to decrease with 4.65% PCE at 140 °C. The enhanced efficiency is attributed to the improved FF as a result of increasing the annealing temperature up to 100 °C. This improvement in FF below 100 °C can be attributed to that PANI and perovskite films become smoother and more homogenous by increasing annealing temperature of PANI, thus decrease trapping of carriers and provide better charge transport as shown by transient photocurrent measurements Fig. S3.c, SI and table S1, SI. In addition, the absence of any solvent residues in the PANI film that may degrade the perovskite film. The decrease in PCE beyond 100 °C is attributed the decrease in Jsc since PANI's conductivity decreases by increasing annealing temperature as a result of degradation of the polymer chain as shown in Fig. S2.d, SI [1].

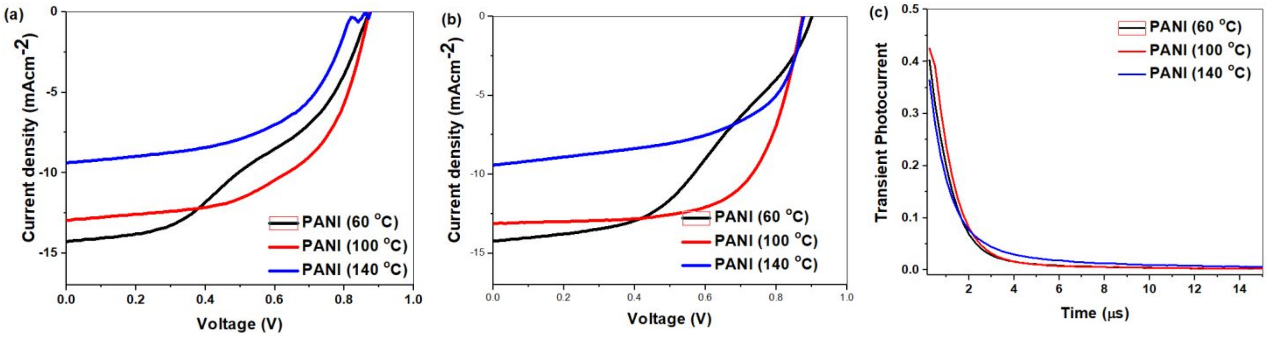

Fig. S3. J-V characteristics (a) Forward and (b) Reverse of perovskite solar cells fabricated using PANI annealed at different temperatures as HTM. (c) Transient photocurrent of perovskite solar cells fabricated using PANI annealed at different temperatures as HTM [1].

Table S1. Photovoltaic parameters of perovskite solar cells fabricated using PANI annealed at different temperatures as HTM [1].

Annealing temp. (°C)	Scan direction	Jsc (mAcm^{-2})	Voc (V)	FF	PCE (%)	Charge carrier transport time (µs)
**60**	Forward	14.28	0.87	0.41	5.12	1.03
	Reverse	14.23	0.91	0.45	5.81	
**100**	Forward	12.97	0.87	0.57	6.39	1.10
	Reverse	13.11	0.88	0.66	7.60	
**140**	Forward	9.38	0.87	0.52	4.21	1.23
	Reverse	9.42	0.88	0.56	4.65	

Effect of HNO_3 doping degree of polyaniline on PSC performance

AFM measurements of PANI doped with different concentrations of HNO_3 (0.8, 0.9, 1.0M) were carried out and their topographical images are shown in fig. S4, SI. The PANI film doped with 0.8M HNO_3 has rms value of 8.00 nm. Increasing HNO_3 concentration to 0.9M decreases rms roughness to 3.35 nm, while further increase to 0.9M HNO_3 increases rms roughness to 21.50 nm. Since HNO_3 act as a dopant as well as a catalyst for polymerization, adding small amount of HNO_3 (0.8M) leads to lower polymerization rate, which eventually forms some PANI-covered regions while some parts on ITO surface are not covered, thus forming a rough PANI film. The increase in roughness for beyond 0.9M HNO_3 is attributed to the enhancement in polymerization rate resulting in agglomeration of PANI and thus increase in film roughness [1].

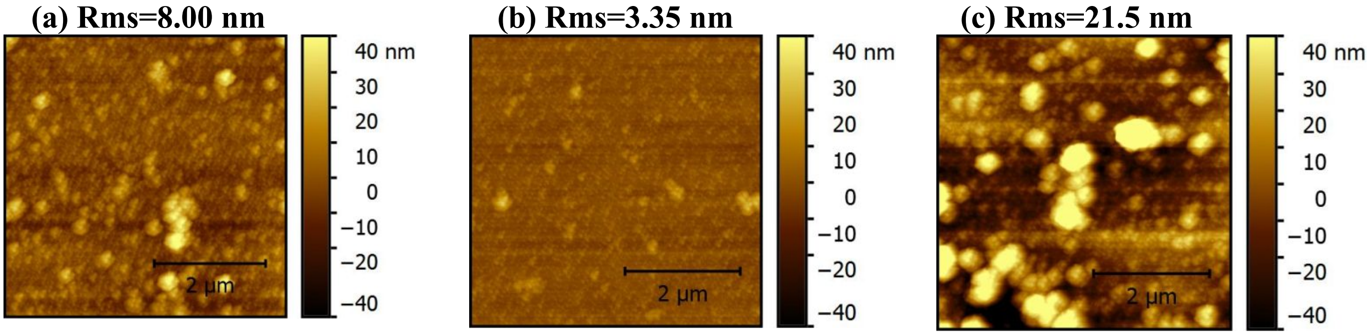

Fig. S4. Tapping mode AFM topography images of electrochemically synthesized HNO_3-PANI doped with (a) 0.8M HNO_3, (b) 0.9M HNO_3, (c) 1.0M HNO_3 [1].

J-V characteristics of PSCs based on PANI doped with different concentrations of HNO_3 as HTM compared with PEDOT:PSS based PSC are shown in Figures S5a,b, SI. Table S2, SI summarizes the photovoltaic performance parameters of the fabricated devices. Results show that the device made of PANI doped with 0.8M HNO_3 achieved PCE up to 7.61%. Increasing the doping degree to 0.9M HNO_3 enhances the PCE to 16.19% which is comparable to that of the PEDOT:PSS based cell with 15.11%. The enhanced PCE is attributed to the improved J_{SC} and FF as a result of increasing the doping degree. This is because the protonation degree of imine groups increases with HNO_3 doping concentration, so conductivity increases. The enhanced J_{SC} is also attributed to the decrease in PANI roughness as shown by AFM results. While further increasing the HNO_3 concentration to 1.0M, PCE decreases to 10.46%. The observed decrease in J_{SC} for beyond 0.9M HNO_3 is because of the increase in roughness which results from the high polymerization rate. This roughness leads to HTM films with gaps that can trap charge carriers, thus depressing charge transport and decreasing J_{SC}. The decrease in Jsc as a result of increasing doping degree to 1.0M is also attributed to protonation of some of the amine groups [1].

Figures S5c,d, SI show transient photocurrent (TPC) and transient photovoltage (TPV) data of perovskite solar cells based on PEDOT:PSS and the PANI films doped with different HNO_3 concentrations, and Table S2, SI shows the obtained values of charge carrier transport time and lifetime. Charge transport times of 1.08 µs, 0.86 µs and 1.34 µs were obtained for the cells fabricated with PANI doped with 0.8M, 0.9M, and 1.0M HNO_3, respectively, compared to a charge transport time of 0.99 µs for the cells with pristine PEDOT:PSS. These results match with JV characteristics (Figure S5a,b, SI) as they confirm the positive effect of doping degree on the charge extraction by the hole transporting PANI and consequently on J_{SC} of the perovskite solar cell. Transient photovoltage (TPV) results are in good agreement with V_{OC} from JV characteristics. The carrier lifetime of PEDOT:PSS, which has the highest V_{OC} (0.97 V), is longest (1.02 µs) compared to 0.72 µs for PANI doped with 0.8M HNO_3. Increasing the doping degree to 0.9M HNO_3 decreased carrier lifetime to 0.53 µs. Further increasing HNO_3 concentration to 1.0M results in a decrease in carrier lifetime to 0.3 µs, thus decreasing V_{OC} from 0.87V to 0.85V (Table S2, SI). This decrease in carrier lifetime and Voc may be because of the shift of the PANI polaron band to a lower work function as a result of increasing doping degree of PANI (emeraldine salt) with HNO_3. Also, the decrease in work function increases the driving

force of hole extraction and collection in the hole transport layer, resulting in shorter transport time and better J_{SC} of the devices [1].

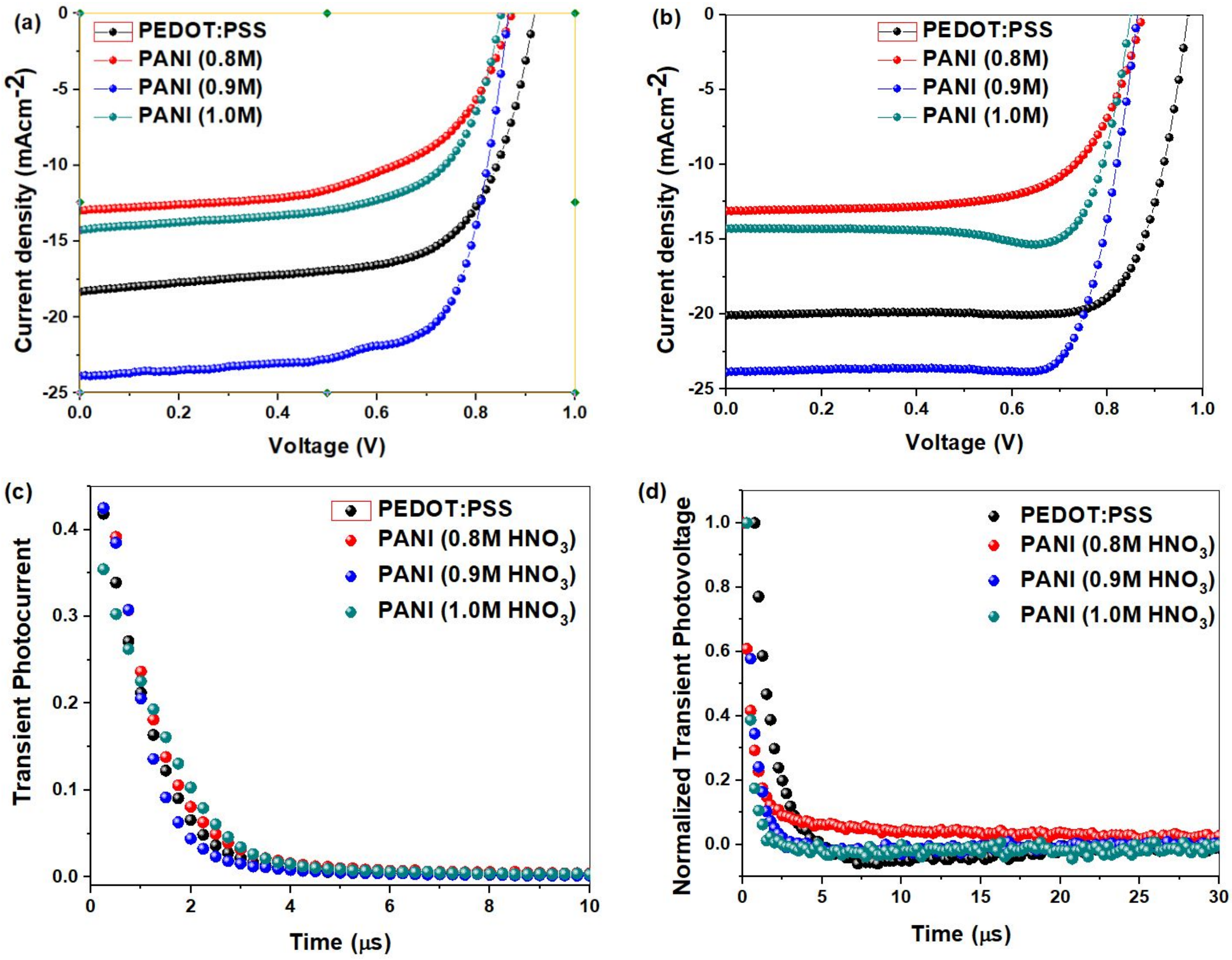

Fig. S5. (a) Forward and (b) reverse J-V characteristics, (c) TPC, and (d) TPV of PSCs made from PEDOT:PSS, and PANI doped with different concentrations of HNO_3 [1].

Table S2. Photovoltaic parameters and charge carrier dynamics parameters of perovskite solar cells made with PEDOT:PSS and PANI doped with different concentrations of HNO_3 as HTMs [1].

**HTM**	**Scan direction**	**J_{SC} ($mAcm^{-2}$)**	**V_{OC} (V)**	**FF**	**PCE (%)**	**Charge carrier transport time (μs)**	**Charge carrier lifetime (μs)**
**PEDOT:PSS**	Forward	18.31	0.92	0.65	11.02	0.99	1.02

	Reverse	20.08	0.97	0.78	15.11		
**PANI ($0.8M\ HNO_3$)**	Forward	12.97	0.87	0.57	6.39	1.08	0.72
	Reverse	13.11	0.88	0.66	7.61		
**PANI ($0.9M\ HNO_3$)**	Forward	23.88	0.87	0.70	14.54	0.86	0.53
	Reverse	23.86	0.87	0.78	16.19		
**PANI ($1.0M\ HNO_3$)**	Forward	14.27	0.85	0.64	7.71	1.34	0.30
	Reverse	15.35	0.85	0.8	10.46		

Effect of LiTFSI doping degree of PANI-HNO_3 on PSC performance

PANI HOMO-LUMO measurement by cyclic voltammetry

The highest occupied molecular orbitals (HOMOs) and the lowest unoccupied molecular orbitals (LUMOs) of the electrochemically synthesized Polyaniline (HNO_3-PANI) were measured using cyclic voltammetry (CV) experiment, carried out on a potentiostat/galvanostat (Ametek VERSASTAT3-200). A solution of 0.1 M tetrabutylammonium hexafluorophosphate (Bu4NPF6) in acetonitrile as electrolyte, platinum wire (Sigma Aldrich) as counter electrode, thin film of HNO_3-PANI deposited on ITO substrates as working electrode and Ag/AgCl as reference electrode were used for the measurements. Voltage was swept at a scan rate of 25 mVs^{-1} at room temperature and in nitrogen gas environment. Ferrocene was used as an external reference. Figure S6a shows one oxidation peak of ferrocene at the potential 0.3V while the electrochemically synthesized HNO_3-PANI has one oxidation peak at the potential 0.9V (fig. S6c) and one reduction peak at the potential -0.9V (fig. S6c). Figure S6d shows two oxidation peaks of electrochemically synthesized HNO_3-LiTFSI-PANI in the positive scan at the potentials 0.9V and 1.0V and fig. S6e shows two reduction peaks in the negative scan at the potentials -0.9V and -1.2V. HOMO and LUMO values were calculated using the equations (1) and (2) [1].

$$\mathbf{E_{HOMO(Polymer)} = E_{HOMO(Ferrocene)} - E_{OX(Polymer)} + E_{OX(Ferrocene)}} \quad (1)$$

$$\mathbf{E_{LUMO(Polymer)} = E_{HOMO(Ferrocene)} - E_{RED(Polymer)} + E_{OX(Polymer)}} \quad (2)$$

Table S3 shows the HOMO and LUMO of HNO_3-PANI with the values -5.4 eV and -3.0 eV, respectively thus the bandgap is 2.4 eV. This large bandgap of HNO_3-PANI makes it transparent to light, thus suitable as a HTM for the pin perovskite solar cell. Doping the electrolyte solution with LiTFSI results in two HOMO values for HNO_3-LiTFSI-PANI (-5.4 eV and -5.5 eV), as well as two LUMO values (-2.6 eV and -3.0 eV) [1].

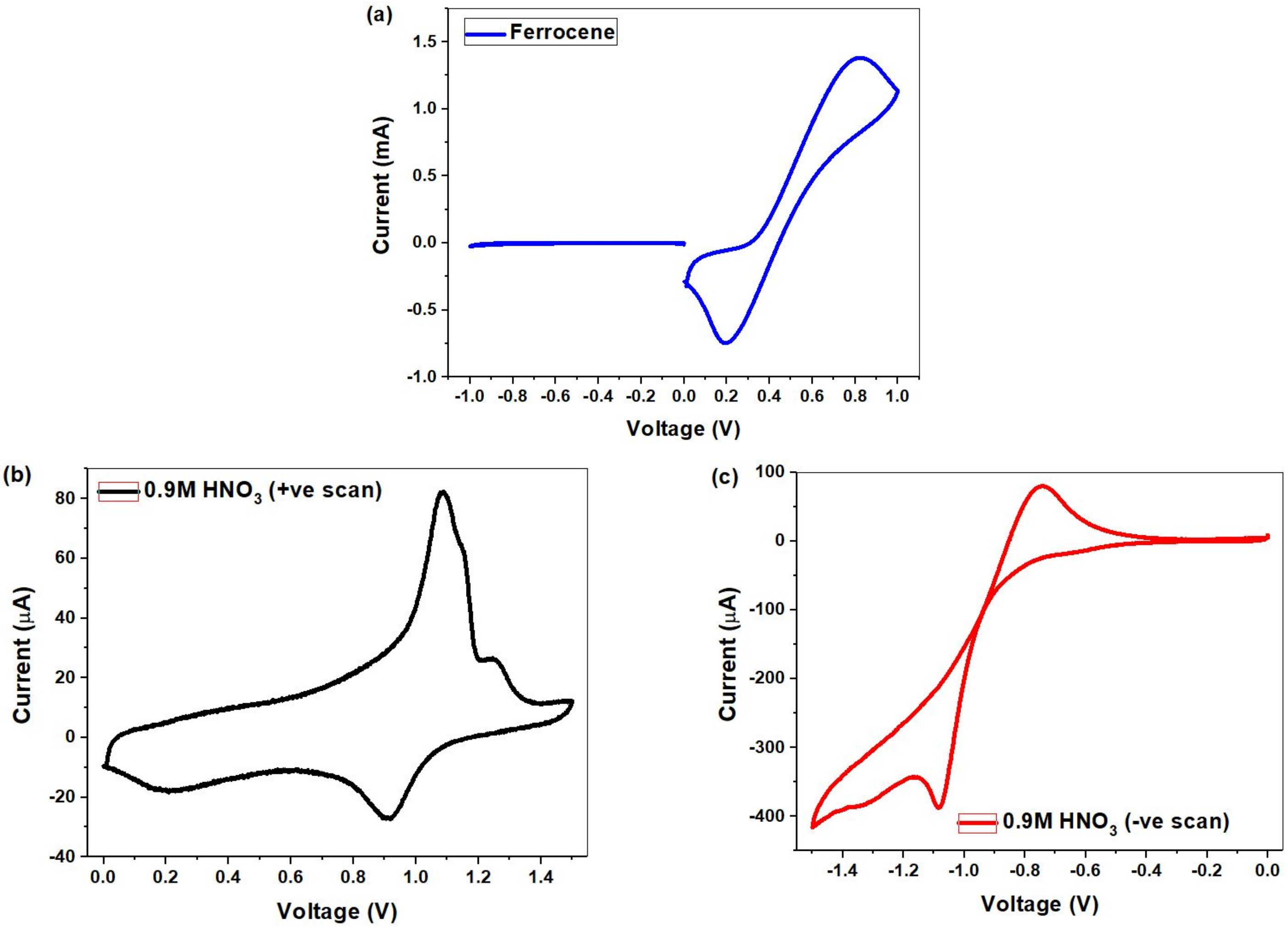

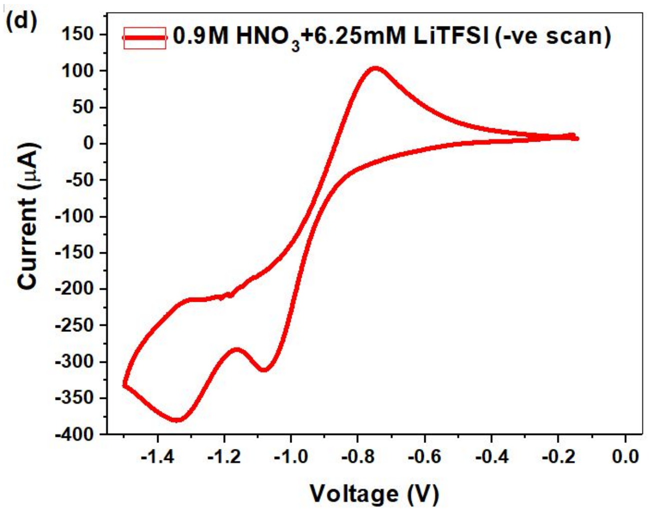

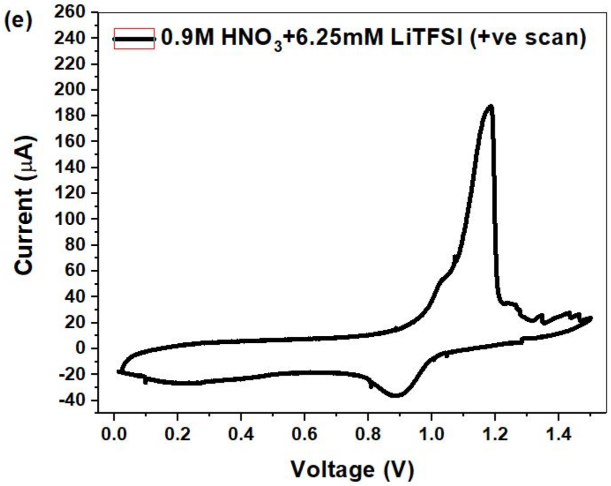

Fig. S6. Cyclic voltammetry (CV) analysis for measuring (a) oxidation potential of ferrocene, (b) HOMO and (c) LUMO of the electrochemically synthesized HNO_3-PANI; (d) HOMO and (e) LUMO of the electrochemically synthesized HNO_3-LiTFSI-PANI [1].

Table S3. Obtained energy level parameters from the Cyclic voltammetry measurements for Ferrocene and electrochemically synthesized HNO_3-PANI with and without LiTFSI [1].

**Material**	**E_{OX} (V)**	**E_{RED} (V)**	**E_{HOMO}**	**E_{LUMO}**
Ferrocene	0.3	----	-4.8	----
HNO_3-PANI	0.9	-0.9	-5.4	-3.0
LiTFSI-HNO_3-PANI	1.0	-1.2	-5.5	-2.6
	0.9	-0.9	-5.4	-3.0

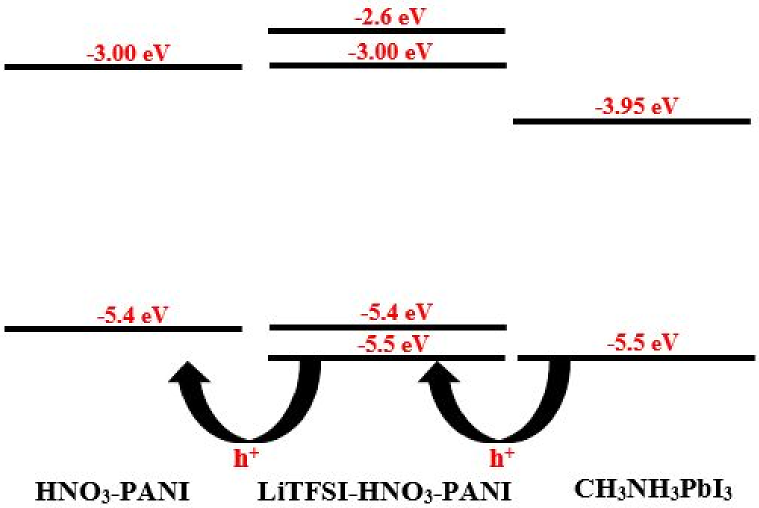

Fig. S7. Schematic diagram showing energy levels of $CH_3NH_3PbI_3$, HNO_3-PANI, and LiTFSI-HNO_3-PANI [1].

AFM measurements of HNO_3-PANI doped with different concentrations of LiTFSI (6.25, 15, 50mM) were carried out, and their topographical images are shown in Figure S8. The HNO_3-PANI film doped with 6.25mM LiTFSI has a Rms roughness value of 2.35nm. Beyond 6.25mM LiTFSI, the rms roughness values increase gradually, which means roughness increases resulting in devices with lower Jsc values. This increase in roughness may be due to excess LiTFSI, blocking PANI from proper polymerization in addition to its agglomeration in large amounts on the PANI surface and between its chains, thus increasing its roughness [1].

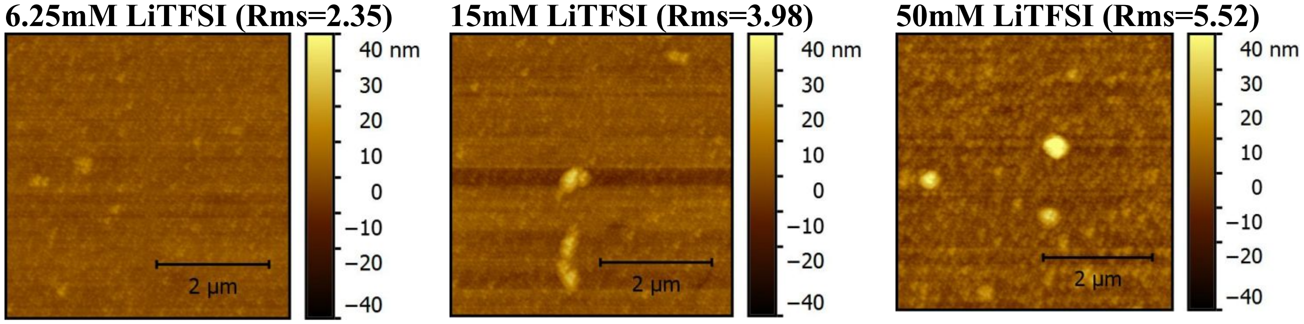

Fig. S8. AFM topography images of HNO_3-PANI doped with different concentrations of LiTFSI (6.25, 15, 50mM).

J-V characteristics of PSCs made of PANI doped with different concentrations of LiTFSI in addition to 0.9M HNO_3 as HTM are shown in Figures S9a,b and Table S4 summarizes the photovoltaic parameters. Results show that the device made of PANI un-doped with LiTFSI salt achieved a PCE of 16.19%. Adding a small amount (6.25mM) LiTFSI into the electrolyte solution enhanced efficiency up to 16.94% as a result of the improved J_{SC} and V_{OC}. This is because doping with LiTFSI increased the work function of PANI (Figure 4), thus increasing V_{OC}. Adding a new energy level in between perovskite and the HTL (PANI) by doping with LiTFSI improved charge extraction at the perovskite/PANI interface, thus enhancing J_{SC}. Also, the improved J_{SC} is because LiTFSI doping decreased the roughness of PANI film as shown by AFM (Figure 2), resulting in PANI film with less gaps, better charge transport across the film and improved J_{SC} [1].

Figures S9c,d show transient photocurrent (TPC) and transient photovoltage (TPV) measurements of perovskite solar cells based HNO_3-PANI films doped with different LiTFSI concentration as HTMs, and Table S4 shows the obtained values of charge carrier transport time and lifetime. Charge transport times of 86 μs, 81 μs, 1.36 μs and 1.49 μs were obtained for the cells fabricated with PANI doped with 0mM, 6.25mM, 15mM and 50mM LiTFSI, respectively [1]. These results match with J_{SC} results from the JV characteristics (Figure S9a,b). Carrier lifetime of the device made of PANI doped with 0mM, 6.25mM, 15mM, 50mM LiTFSI had values in the order of 0.53 μs < 0.75 μs < 0.82 μs < 1.25 μs, respectively. These results are in good agreement with the V_{OC} from the JV characteristics which showed that V_{OC} of the device made of PANI doped with 0mM, 6.25mM, 15mM, 50mM LiTFSI had values in the order of 0.87V < 0.93V < 0.95V < 0.96V respectively [1].

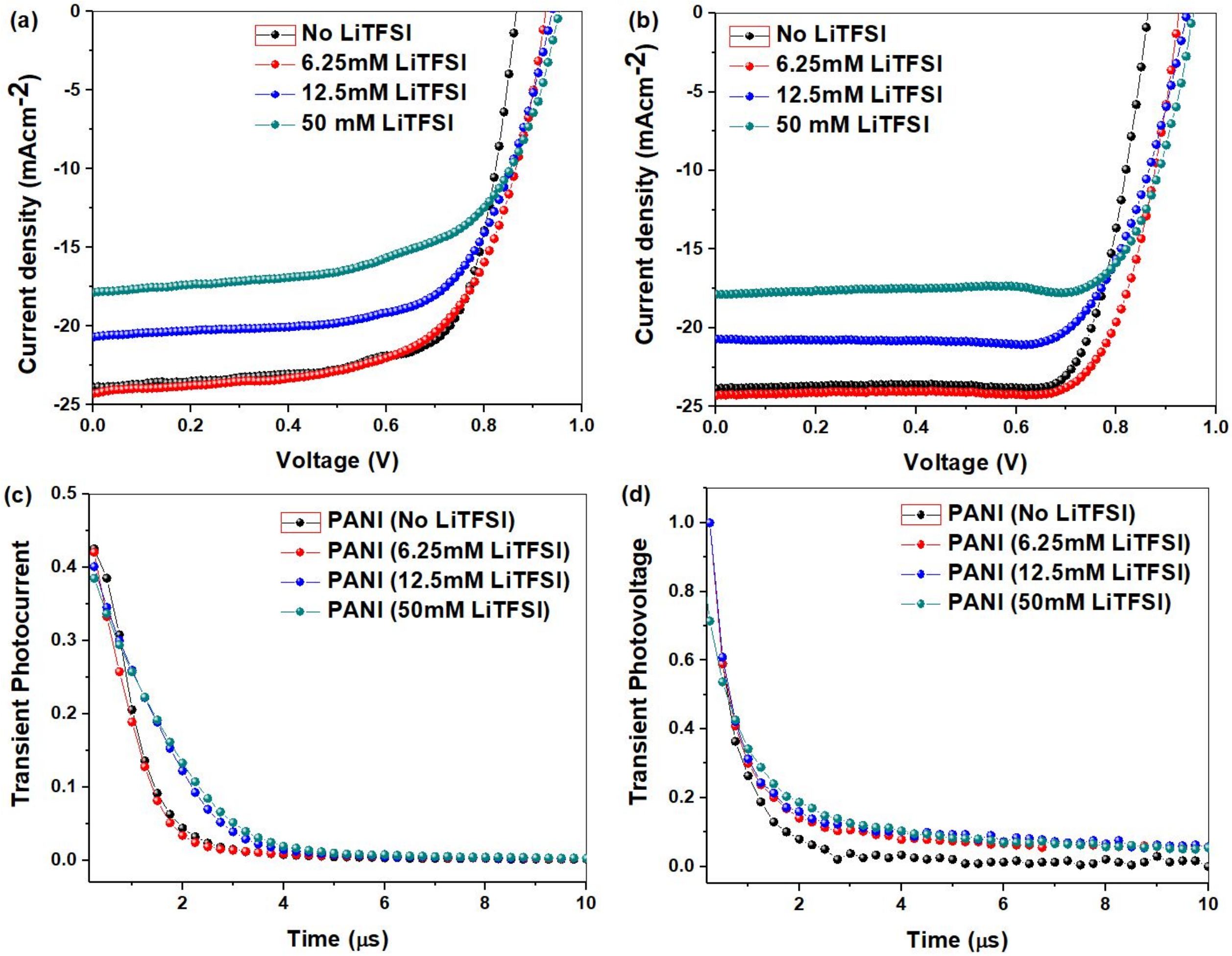

Fig. S9. (a) Forward and (b) reverse J-V characteristics, (c) TPC, and (d) TPV measurements of PSCs made from PANI doped with different concentrations of LiTFSI [1].

Table S4. PV parameters and transient data for PSCs made from PANI doped with different concentrations of LiTFSI [1].

**Concentration of LiTFSI (mM)**	**Scan direction**	**J_{SC} ($mAcm^{-2}$)**	**V_{OC} (V)**	**FF**	**PCE (%)**	**Charge carrier transport time (μs)**	**Charge carrier lifetime (μs)**
0	Forward	23.88	0.87	0.70	14.54	0.86	0.53
	Reverse	23.86	0.87	0.78	16.19		

6.25	Forward	24.28	0.93	0.63	14.23	0.81	0.75
	Reverse	24.28	0.93	0.75	16.94		
12.5	Forward	20.68	0.94	0.65	12.62	1.36	0.82
	Reverse	21.08	0.95	0.71	14.13		
50	Forward	17.85	0.95	0.61	10.36	1.49	1.25
	Reverse	17.87	0.96	0.76	12.95		

Kelvin Prop Force Microscope

Energy levels of HTL need to be well matched with that of the $CH_3NH_3PbI_3$ perovskite to facilitate efficient hole transfer. To investigate the barrier of back recombination at the perovskite/HTM interface and within the particles of the hole transport material, kelvin probe force microscopy (KPFM) was measured together with AFM which uses a conducting tip as a Kelvin probe to measure the surface potential. When the semiconducting material (sample) and the tip come in contact, contact potential difference (E_{CPD}) will exist between them, and electrons will flow from the material with lower work function (E_{FS}) to that with the higher work function (E_{FT}), and there will be electrostatic force between them. This electrostatic force is nullified by applying an external bias. When the external bias equals the CPD, the work function of the sample can be estimated using the equation [3-4]:

$$V_{CPD} = \frac{\emptyset_{Tip} - \emptyset_{Sample}}{-e}$$

where $\emptyset_{Tip}$ and $\emptyset_{Sample}$ are the work functions of the tip and sample, respectively [1].

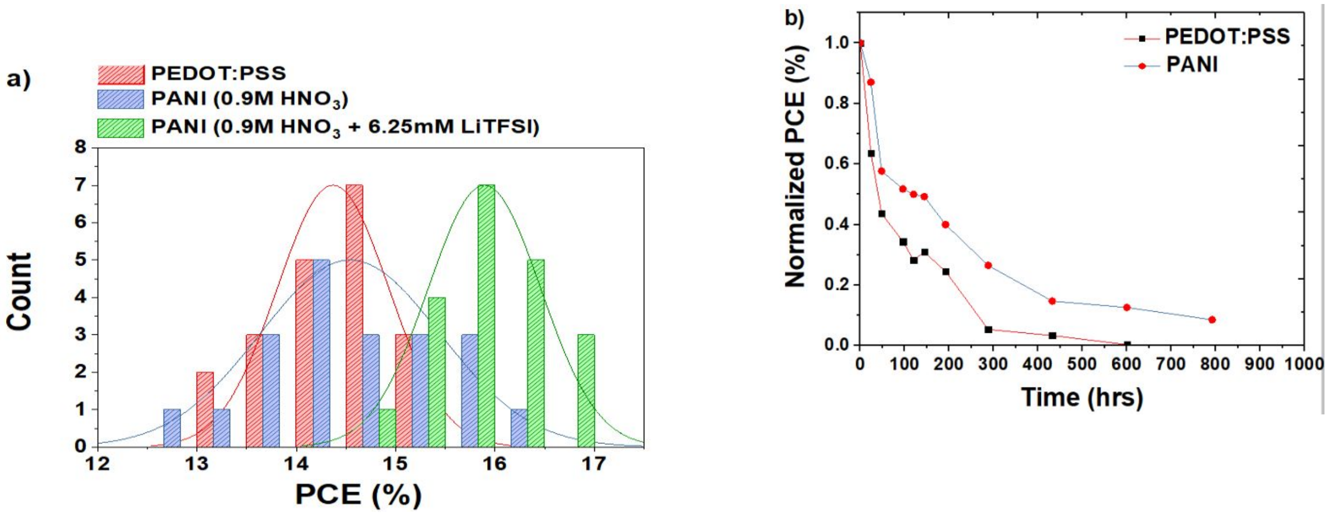

Fig. S10 (a) Histogram of PCE from 20 individual perovskite solar cells based on PEDOT:PSS and the optimized PANI HTLs. (b) Stability test of p-i-n perovskite solar cells based on PEDOT:PSS and PANI hole transport layers at ambient condition 23±1 °C and 50±5 % humidity.

1. Mabrouk, S. Engineering of Hole Transport and Perovskite Absorber Layers to Achieve High Efficiency and Stable Perovskite Solar Cells. Ph.D., South Dakota State University, Ann Arbor, 2018.
2. Mabrouk, S.; Bahrami, B.; Elbohy, H.; Reza, K. M.; Gurung, A.; Liang, M.; Wu, F.; Wang, M.; Yang, S.; Qiao, Q., Synergistic engineering of hole transport materials in perovskite solar cells. *InfoMat* **2020,** *2* (5), 928-941.
3. Melitz, W.; Shen, J.; Kummel, A. C.; Lee, S., Kelvin probe force microscopy and its application. *Surf. Sci. Rep.* **2011,** *66* (1), 1-27.
4. Rosenwaks, Y.; Shikler, R.; Glatzel, T.; Sadewasser, S., Kelvin probe force microscopy of semiconductor surface defects. *Phys. Rev. B* **2004,** *70* (8), 085320.
